# Examining the Effectiveness of Social Media for the Dissemination of Research Evidence for Health and Social Care Practitioners: Systematic Review and Meta-Analysis

**DOI:** 10.2196/51418

**Published:** 2024-06-05

**Authors:** Sarah Roberts-Lewis, Helen Baxter, Gill Mein, Sophia Quirke-McFarlane, Fiona J Leggat, Hannah Garner, Martha Powell, Sarah White, Lindsay Bearne

**Affiliations:** 1 Population Health Research Institute St George’s University of London London United Kingdom; 2 Bristol Population Health Science Institute University of Bristol Bristol United Kingdom; 3 National Institute of Health and Care Research London United Kingdom; 4 Faculty of Health, Social Care and Education St George's University of London London United Kingdom; 5 School of Psychology University of Surrey Guildford United Kingdom; 6 Department of Physiotherapy St George's University Hospitals NHS Foundation Trust London United Kingdom

**Keywords:** social media, dissemination, health care, social care, research evidence, practitioners, effectiveness, meta-analysis, systematic review, randomized controlled trial, RCT

## Abstract

**Background:**

Social media use has potential to facilitate the rapid dissemination of research evidence to busy health and social care practitioners.

**Objective:**

This study aims to quantitatively synthesize evidence of the between- and within-group effectiveness of social media for dissemination of research evidence to health and social care practitioners. It also compared effectiveness between different social media platforms, formats, and strategies.

**Methods:**

We searched electronic databases for articles in English that were published between January 1, 2010, and January 10, 2023, and that evaluated social media interventions for disseminating research evidence to qualified, postregistration health and social care practitioners in measures of reach, engagement, direct dissemination, or impact. Screening, data extraction, and risk of bias assessments were carried out by at least 2 independent reviewers. Meta-analyses of standardized pooled effects were carried out for between- and within-group effectiveness of social media and comparisons between platforms, formats, and strategies. Certainty of evidence for outcomes was assessed using the GRADE (Grading of Recommendations, Assessment, Development, and Evaluations) framework.

**Results:**

In total, 50 mixed-quality articles that were heterogeneous in design and outcome were included (n=9, 18% were randomized controlled trials [RCTs]). Reach (measured in number of practitioners, impressions, or post views) was reported in 26 studies. Engagement (measured in likes or post interactions) was evaluated in 21 studies. Direct dissemination (measured in link clicks, article views, downloads, or altmetric attention score) was analyzed in 23 studies (8 RCTs). Impact (measured in citations or measures of thinking and practice) was reported in 13 studies. Included studies almost universally indicated effects in favor of social media interventions, although effect sizes varied. Cumulative evidence indicated moderate certainty of large and moderate between-group effects of social media interventions on direct dissemination (standardized mean difference [SMD] 0.88; *P*=.02) and impact (SMD 0.76; *P*<.001). After social media interventions, cumulative evidence showed moderate certainty of large within-group effects on reach (SMD 1.99; *P*<.001), engagement (SMD 3.74; *P*<.001), and direct dissemination (SMD 0.82; *P*=.004) and low certainty of a small within-group effect on impacting thinking or practice (SMD 0.45; *P*=.02). There was also evidence for the effectiveness of using multiple social media platforms (including Twitter, subsequently rebranded X; and Facebook), images (particularly infographics), and intensive social media strategies with frequent, daily posts and involving influential others. No included studies tested the dissemination of research evidence to social care practitioners.

**Conclusions:**

Social media was effective for disseminating research evidence to health care practitioners. More intense social media campaigns using specific platforms, formats, and strategies may be more effective than less intense interventions. Implications include recommendations for effective dissemination of research evidence to health care practitioners and further RCTs in this field, particularly investigating the dissemination of social care research.

**Trial Registration:**

PROSPERO International Prospective Register of Systematic Reviews CRD42022378793; https://www.crd.york.ac.uk/prospero/display_record.php?RecordID=378793

**International Registered Report Identifier (IRRID):**

RR2-10.2196/45684

## Introduction

### Background

It is essential that health and social care practitioners access contemporary, high-quality research evidence to help them deliver the best evidence-based clinical care and improve patient outcomes [1-5]. Rapid dissemination, by active approaches using specific channels and planned strategies, is recommended [6,7].

Social media may facilitate rapid dissemination to busy practitioners, allowing them to access and interpret research evidence efficiently [8-10]. Because social media are widely used and not limited in space and time [8,11], they have the potential to overcome barriers to dissemination, including reaching practitioners with limited professional opportunities or time constraints and filtering the exponentially increasing volume of research evidence produced every year [9,12-14]. Currently, closed social media channels, such as private and invitation-only groups, are often used by practitioners for day-to-day communications, clinical information sharing, and targeted clinical education, whereas open social media channels that can be accessed by everybody are used for reputation development; public health education; and, increasingly, research dissemination [9,10,15-26].

However, the effectiveness of open social media for the dissemination of research evidence to health and social care practitioners is largely unknown [27]. Existing reviews have narratively synthesized potential uses, benefits and risks, similarities and differences, and qualitative experiences of social media or provided commentaries on the mechanisms of research dissemination by social media [10,22,23,28]. No reviews have conducted a meta-analysis to quantitatively test the effectiveness of social media for the dissemination of research evidence to health and social care practitioners. To inform evidence-based recommendations, the evidence for using social media to disseminate research evidence must be investigated.

### Objectives

The primary research question was as follows: “How effective is open social media as a way to disseminate research evidence to practitioners?” The objective of this systematic review was to quantitatively synthesize and meta-analyze evidence of the effectiveness of social media for the dissemination of research evidence to health and social care practitioners by evaluating both between-group comparisons of social media versus no social media and within-group comparisons of before-after social media campaigns. The social media platforms, formats, and strategies used were also identified, and their effectiveness was compared to understand the most effective social media intervention characteristics for the dissemination of research evidence to practitioners.

## Methods

### Design

The protocol was registered on the International Register of Systematic Review (PROSPERO; CRD42022378793) and published a priori [29]. It was reported in accordance with the PRISMA (Preferred Reporting Items for Systematic Reviews and Meta-Analyses) guidance [30] (Multimedia Appendix 1).

### Eligibility Criteria

Articles published between January 1, 2010, and January 10, 2023, were eligible for inclusion if they investigated research evidence targeted at health and social care practitioners that was shared using open social media. Articles were included if they quantitatively compared social media versus no social media (either between-group comparisons or before-after social media within-group comparisons) or if they compared social media platforms, formats, or strategies. Eligible study designs included randomized controlled trials (RCTs), case-controlled comparisons, crossover, nonrandomized group comparisons, before-after comparisons, cohort comparisons, and case reports. Eligible outcomes of interest included reach, engagement, direct dissemination, and impact. Definitions of the eligibility criteria terms are shown in Textbox 1.

Articles were not eligible for inclusion if they only compared social media effectiveness in terms of the topic or specialty of the research evidence–related social media post or posts. Excluded study designs included protocols, reviews, studies using only qualitative methods, opinion pieces, and conference abstracts with no linked full-text article. Articles were excluded if they preceded 2010 (refer to the protocol by Roberts-Lewis et al [29]), were not available in English, or did not feature research evidence–related social media of relevance to postregistration health or social care practitioners (eg, only targeting students, service users, or the public or featuring non–health and social care research topics). Articles were also excluded if the social media campaign was targeted at practitioners for purposes other than the dissemination of research evidence (eg, delivering multisource clinical education courses, organizational information, administrative tasks, practical peer support, day-to-day interpersonal clinical communication, professional identity, or reputation promotion). Finally, articles that did not provide sufficient quantitative empirical data on reach, engagement, direct dissemination, or impact were excluded.

**Definitions of the eligibility criteria terms.**

**Definitions**

- Research evidence: this was defined as published, peer-reviewed empirical human health and social care research findings that have met the publication standards of their specialty, presented as an original research article (primary research), a group of original research articles identified and synthesized systematically (secondary research), or evidence drawn together for evidence-based guidelines or clinical recommendations. Where research evidence was posted on social media, it either included a direct link to an open-access article or research information that had been summarized in the form of abstracts, microblogs, blogs, press articles, infographics, or educational videos.
- Targeted: by this we mean research evidence or social media posts that were professionally relevant to health and social care practitioners. Evidence was eligible if it was produced specifically for practitioners or when evidence was relevant to practitioners but other audiences such as the public also had access.
- Practitioners: these were postregistration health and social care professionals, collectively or as individual professions including but not limited to nurses, doctors, social workers, midwives, pharmacists, physiotherapists, occupational therapists, radiographers, and paramedics.
- Open social media: we defined open social media as internet-based social networking and media sharing platforms that allow any user to create and exchange user-generated content, making one-to-many posts and interacting by responding to others’ posts. Our definition did not include mass media press articles, wikis, and blogs with no or limited facility for user interactions. Our definition also did not include purely communication-based apps, fee-paying, or closed, invite-only social media groups that could not be freely joined by any interested user. However, both noninteractional and closed social media groups were considered within our definition if they were highlighted on, or accessible via, open social media.
- Platforms: these were defined as open social networking and media sharing sites and apps, including but not limited to Facebook, YouTube, Instagram, WeChat, Tumblr, TikTok, Reddit, Twitter (subsequently rebranded X), and LinkedIn.
- Formats: these were a variety of media types, including but not limited to text, illustrative pictures, visual abstracts, infographics, videos, and podcasts.
- Strategies: these were the ways in which research evidence–related social media posts were delivered, including but not limited to a schedule of open sharing to the entire forum (frequency and timing), influencer endorsement, @mentions and #tagging, accessible special interest groups (eg, journal clubs), and live social media events (eg, tweet chats).
- Reach: this was defined as the number of practitioners reached by research evidence–related social media post or posts (eg, those following the social media account or participating in a social media event) or the social media analytics including the number of impressions (the number of times a post appears on social media feeds), views (the number of times a post is opened from social media feeds), or accesses (the number of times a post is accessed in any other way, eg, via a search engine).
- Engagement: this was measured by the number of positive responses (ie, likes) or interactions (such as shares, comments, reposting, or new posts) generated by a research evidence–related social media post.
- Direct dissemination: this was measured by the number of times an original piece of research evidence was accessed (eg, by link click from a social media post), viewed (eg, on an HTML web page), downloaded (eg, as a PDF document) or the altmetric attention score accumulated by original research articles.
- Impact: this included two discrete subcategories for the purposes of this review—(1) academic impact, the number of citations received by an original research evidence article or the journal impact factor, and (2) practical impact, measures of practitioners’ changes in thinking or practice (eg, confidence, knowledge, or behavior change) after exposure to research evidence–related social media post or posts.

### Information Sources

Six electronic databases were searched (MEDLINE [Ovid], PsycINFO [Ovid], CINAHL plus [EBSCO], and ERIC [EBSCO] as well as LISTA and OpenGrey). The date of the last search was January 10, 2023. Bibliographies of relevant reviews and included articles were searched for citations and PubMed, Elicit, and Google Scholar were used for reference harvesting.

### Search Strategy

For full search strategies, refer to Multimedia Appendix 2 and the protocol by Roberts-Lewis et al [29]. Key search terms were grouped as follows:

- Practitioner groups, for example, health and social care staff and individual disciplines
- Research evidence, information, and knowledge
- Social media, network, web, sharing, and named platforms and formats
- Dissemination, reach, engagement, and impact
- Quantitative, evaluation, comparison, and named outcomes

### Selection Process

Records from the electronic and citation searches were exported to EndNote Online (Clarivate) for deduplication and then imported to Rayyan software (Rayyan Systems) for title, abstract, and full-text screening.

Title and abstract screening were carried out by 2 independent reviewers (SRL and SQM). There was 92% agreement (κ=0.87) on eligibility decisions and 100% agreement after discussion.

Full-text screening was carried out by at least 2 of 5 independent reviewers (SRL, SQM, LB, HG, and FJL). There was 84% agreement (κ=0.83) on full-text inclusion decisions and 100% agreement after discussion.

### Data Collection

Data from the included studies were extracted independently by at least 2 of 5 reviewers (see the *Selection Process* section) using a data extraction form developed a priori [29]. The accuracy of data extraction was confirmed by comparison between extraction forms, returning to the original article to resolve any disparity.

### Data Items

The variables collected were study characteristics including the number and description of subjects; social media platforms, formats, and strategies; study design; comparisons; and outcomes. For each outcome of interest, means, SDs, and sample sizes were extracted for each comparison. When these data were missing, they were calculated from other reported statistics using recommended methods [31], where possible.

For studies that reported multiple outcome measures, only outcomes of interest were collected (reach, engagement, direct dissemination, and impact). Different measures for the same outcome were prioritized for inclusion in meta-analyses according to the a priori protocol [29]. Subsequent additions were made to the prioritization order to account for heterogeneous data reported in the included studies; these included aggregated total interactions, other types of post interactions, and the measurement time frame according to the most common time frames for each outcome of interest (Textbox 2).

**Summary of the outcomes of interest and their prioritization order for entry into meta-analyses.**

**Outcome and prioritizations**

- Reach
  Number of practitioners after 1 week
  (1) Impressions, (2) views, and (3) accesses after 1 month
- Engagement
  Number of positive responses (ie, likes) after 1 week
  Number of post interactions—(1) total interactions (including shares, comments, and other interactions); (2) shares; (3) comments; (4) new posts, and (5) other post interactions—after 1 month
- Direct dissemination
  (1) Link clicks and (2) article views after 1 month
  Article downloads after 1 month
  Altmetric attention score after 1 month
- Impact
  (1) Citations and (2) impact factor after 1 year
  Any measures of thinking or practice after any time frame

### Risk of Bias Assessment

The 34-item (5-domain) Cochrane Risk of Bias 2.0 tool was used to rate the quality of the RCTs as lower risk of bias, some concerns, or higher risk of bias [32]. The Newcastle-Ottawa Scale (score range 0-9) was used to assess the risk of bias in nonrandomized designs [33]. A score of ≤3 was considered high risk of bias, scores between 4 and 6 were considered medium risk of bias, and a score of ≥7 was considered low risk of bias [33]. Risk of bias was assessed independently by at least 2 reviewers and data were checked for accuracy by a third reviewer.

### Data Synthesis

The included studies were summarized narratively in text, tables, and figures. Quantitative comparisons were made using calculated standardized mean differences (SMDs), CIs, and *P* values for each comparison. SMD effect sizes were calculated using Hedges *g* to accommodate the heterogeneity of outcomes. Effect sizes of >0.8 were defined as large, ≥0.5 to 0.8 as moderate, and <0.5 as small [34]. Outcome effect sizes were presented as SMD, 95% CIs, *z*-test, and *P* value.

For outcomes where group means, SDs, and sample sizes were obtained from at least 2 studies, pooled effect sizes were calculated using random effects models in RevMan (version 5.3; The Cochrane Collaboration). The heterogeneity of pooled data was assessed using *I*^2^. Funnel plots were assessed visually for each meta-analysis to check for publication bias.

For pooled data with *I*^2^>75%, subgroup analyses were planned; however, these were not possible due to the heterogeneous characteristics of the social media interventions in the included studies or an insufficient number of studies to achieve ≥80% statistical power [35]. Therefore, studies were ordered according to effect size, and the common characteristics of social media strategies in the studies with the largest effect sizes were narratively synthesized. Although no sensitivity analysis was planned a priori, evidence from RCTs and studies with low risk of bias was given greater weighting in the narrative synthesis than nonrandomized studies and those with high risk of bias.

### Certainty Assessment

For each outcome, the certainty of the evidence base was evaluated based on the GRADE (Grading of Recommendations, Assessment, Development, and Evaluations) approach [36] and categorized as high, moderate, low, or very low [37,38].

## Results

**Figure 1:**
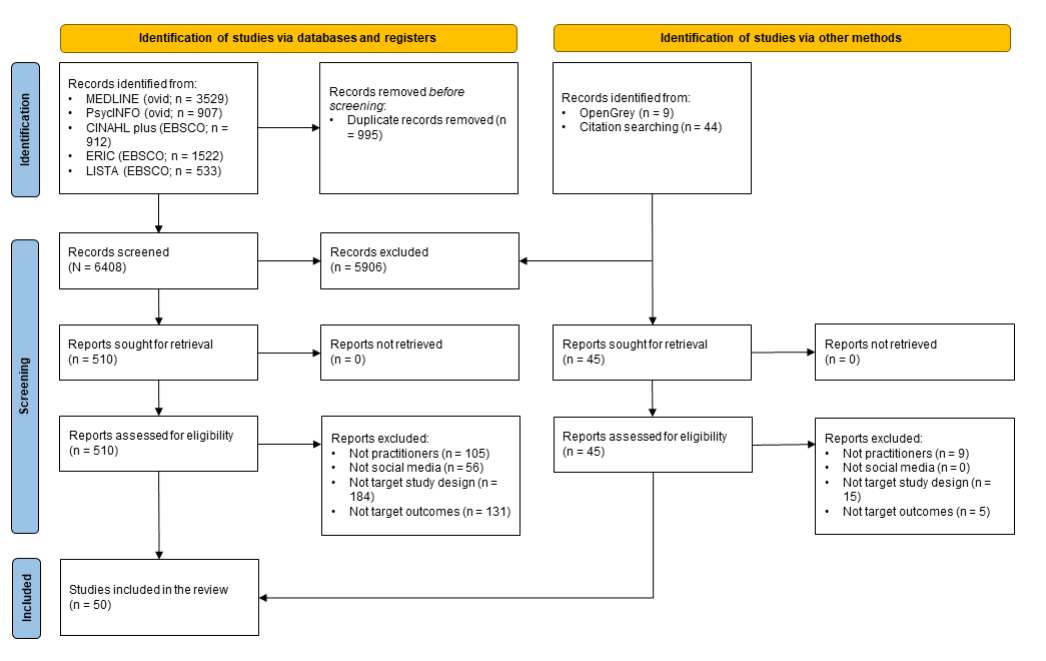
PRISMA (Preferred Reporting Items for Systematic Reviews and Meta-Analyses) flow diagram illustrating the process of study selection for a systematic review on the effectiveness of social media for dissemination of research evidence for health and social care practitioners, detailing a total of 6451 records identified (5896 excluded), 555 full-text reports screened (505 excluded), and 50 articles included.

In total, 6461 records were identified, 555 full-text reports were screened, and 50 articles were included (Figure 1).

### Included Studies

A total of 50 studies published between 2013 and 2022 were included; 9 were RCTs [39-48]. In total, 26 studies included nonrandomized comparisons [49-74], 12 studies were before-after comparisons [75-86], and 3 were case studies [87-89]. A total of 10 studies included both between-group comparisons and before-after analyses [40,44,45,47,51,53,60, 63,70,74]. For study descriptions, refer to Multimedia Appendix 3 [39-89].

A total of 36 studies investigated the impact of social media on journal articles, with samples ranging from a single journal article [88] to 15,078 articles from multiple journals [54]. In total, 8 studies focused on research blogs [40,76,87] and microblogs [47,49,57,82,85], 4 studies examined research conference social media posts and hashtags [71,74,86,89], 2 studies investigated clinical guidelines [60,63], and 1 study tested research-related posts linked to health care hashtags [62].

Half of the studies explored multiple social media platforms and the other half of the studies examined a single platform. Twitter was used in all but 1 study [85], Facebook was used in 23 studies [40,42-44,47,48,53,57,60,63,64,67-70,76,77, 80-82,85,89], LinkedIn was used in 8 studies [40,48,60,63,64,68,87,89], Instagram was used in 6 studies [57,62,67,68,76,87], and YouTube was used in 5 studies [58,60,63,64,87], whereas TikTok [87], Weibo [39], Google+ [64], Tumblr [81], and Spotify [87] were each used in 1 study. The most common media formats examined were text posts, which usually included links and images. Journal clubs or tweet chats were included in 9 studies [51,60,62,74,75,77,78,80,81], and video media were used in 6 studies [52,60,62,63,73,78]. The social media campaign duration ranged from 1 hour [51] to 5.5 years [54].

Outcomes measurement duration ranged from 3 days [86,89] to 4 years [64]. Typically, outcomes were measured after 1 month [42-44,48,52,53,56,60,63,67,75-81,83-85,87] or 1 to 2 weeks [39-41,45,47,51,68,74,82,88]. Citations were measured after ≥1 year, except in 1 study that reported citations after 6 months [61].

### Risk of Bias

In total, 5 RCTs had low risk of bias [39,41-43,45,46], 3 RCTs had some concerns [40,44,48], and 1 RCT had high risk of bias [47] (Multimedia Appendix 3). The most common reasons for risk of bias included insufficient information provided about the allocation sequence, handling of missing data, or prioritization of multiple eligible outcome measurement time points. In total, 11 nonrandomized studies had low risk of bias [49,50,55-57,63,65,70,72,79,81]; 25 nonrandomized studies had moderate risk of bias [51-54,58,59,61,62,64,66-69,71,73-78, 80,82-85]; and 5 nonrandomized studies had high risk of bias [60,86-89] (Multimedia Appendix 3). The most common reasons for risk of bias included targeted selection of studies for social media sharing and incomplete reporting of data handling. Funnel plots did not indicate a high risk of publication bias in pooled data.

### Reach

#### Overview

In total, reach was evaluated in 26 studies (2 RCTs [41,45]). A total of 10 studies evaluated reach by reporting the numbers of practitioners receiving posts [41,45,66,67,75,78,79,84,86,89]. In total, 23 studies evaluated reach using social media analytics (17 in impressions [41,45,49,51,53,56,57,66,68,73,75,78,83, 85,86,88,89]; 6 in views [62,63,76,79,81,87]; and none by reporting accesses).

#### Effects of Social Media Compared to No Social Media on Reach

There were insufficient studies comparing the reach of social media interventions versus no social media for pooled data analyses.

Evidence from individual studies included 1 RCT [45] with a low risk of bias that found a large between-group effect on the number of physicians reached by tweeted articles in a coordinated campaign, including a team with 12 social media accounts, 4 articles tweeted per day, and @mentions of authors and relevant institutions, compared to not tweeted articles (112 cardiothoracic surgery research articles; SMD 4.03, 95% CI 3.37-4.68; *P*<.001). Similarly, 1 nonrandomized study [63] with a low risk of bias reported a large between-group effect on views in favor of YouTube videos marketed by paid social media advertising on Facebook, Twitter, and LinkedIn, with relevant event hashtags, compared to video views in the absence of social media marketing (12 videos about tracheostomy safety; SMD 2.53, 95% CI 1.41-3.64; *P*<.001; Multimedia Appendix 3).

#### Within-Group Effects of Social Media on Reach

**Figure 2:**
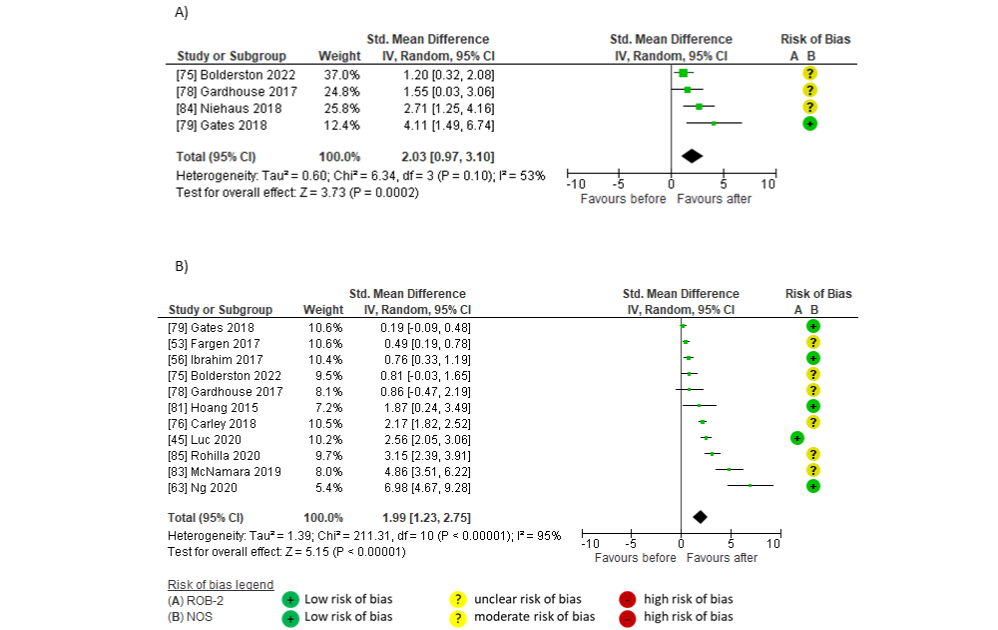
Meta-analyses of within-group effects after social media interventions on reach in (A) the number of practitioners and (B) impressions or post views.

Pooled findings indicated large within-group effects on reach after social media interventions compared to before in both number of practitioners (SMD 2.03, 95% CI 0.97-3.10; *P*<.001; *I*^2^=53%; GRADE moderate) [75,78,79,84] and impressions or views (SMD 1.99, 95% CI 1.23-2.75; *P*<.001; *I*^2^=95%; GRADE moderate) [45,53,56,63,75,76,78,79,81,83,85]. The largest effects were reported in studies featuring social media marketing and scheduling tools [63,83] and multiple social media platforms (Twitter, Facebook, Instagram, and Tumblr) [63,76,81]; at least 1 post per day [76,79,81,83,84], including regular blogs [76,79,81] or microblogs [85]; posts coordinated with established live journal clubs, relevant events, hashtags, and @mentions [45,63,75,78,83]; and campaigns lasting 6 months to 4.5 years [76,81,85]. Smaller effects were reported by studies featuring one-off or less well-established tweet chats or events [75,78], 1 to 2 posts per month [53,56,75,78], and campaigns using a single social media platform (Twitter) [56,75,78,79] (Figure 2 [45,53,56,63,75,76,78,79,81,83-85]).

#### Between-Group Effects of Different Platforms, Formats, and Strategies on Reach

Pooled findings indicated a large between-group effect on impressions and views in favor of Twitter (vs Facebook and Instagram; SMD 1.87, 95% CI 1.54-2.21; *P*<.001; *I*^2^=0%; GRADE low) and Facebook (vs Instagram; SMD 1.19, 95% CI 0.64-1.75; *P*<.001; *I*^2^=46%; GRADE low) [57,68]. However, no effect was shown between platforms in the number of practitioners who were followers on Twitter, Facebook, and Instagram [67] (Multimedia Appendix 3).

Pooled findings showed a large effect on impressions in favor of posts with images, in particular, infographics, compared to no images (SMD 1.63, 95% CI 0.04-3.22; *P*=.04; *I*^2^=95%; GRADE low; Multimedia Appendix 3) [41,45,49,56].

Pooled findings indicated a large effect on reach in favor of strategies using social media influencers or organizations compared to using standard social media user accounts (SMD 1.02, 95% CI 0.04-1.99; *P*=.04; *I*^2^=100%; GRADE low) [66,73]. One RCT follow-up study [46] also reported that tweeting at 1 PM (EST, United States) generated the highest reach and tweeting at 9 PM generated the lowest reach to physicians (Multimedia Appendix 3).

### Engagement

A total of 21 studies (including 3 RCTs [41,45,47]) evaluated engagement (6 studies examined likes [45,47,49,51,83,88]; 11 studies investigated total engagements, including shares, comments, and other interactions [41,45,49,51,53,54, 67,71-73,78]; 7 studies assessed only post shares [47,49,52,56,68,83,84]; and 2 studies reported on other post interactions only [63,75]). No included studies evaluated comments or reposts alone.

#### Effects of Social Media Compared to No Social Media on Engagement

There were insufficient studies comparing the engagement of social media interventions versus no social media for pooled data analyses.

Evidence from individual studies included just 1 nonrandomized study [63] with a low risk of bias that reported a large between-group effect on interaction time spent watching YouTube videos marketed by paid social media advertising compared to video interaction time in the absence of social media marketing (12 videos about tracheostomy safety; SMD 2.36, 95% CI 1.27-3.44; *P*<.001).

#### Within-Group Effects of Social Media on Engagement

**Figure 3:**
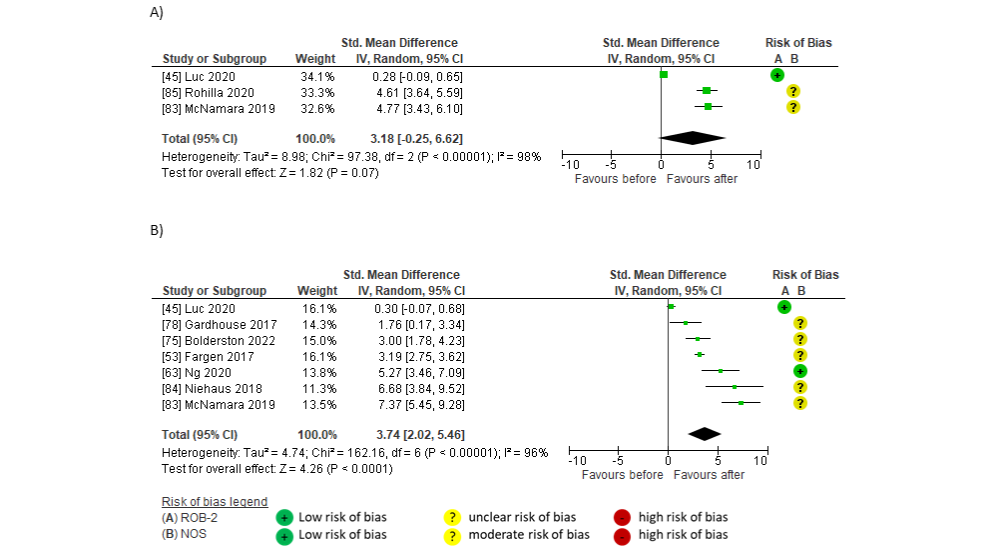
Meta-analyses of within-group effects after social media interventions on engagement in (A) positive responses (likes) and (B) post interactions (total interactions, shares [retweets], comments [replies], or other post interactions).

Pooled findings indicated large within-group effects on engagement after social media interventions compared to before. Effects were significant for post interactions (SMD 3.74, 95% CI 2.02-5.46; *P*<.001; *I*^2^=96%; GRADE moderate) [45,53,63,75,78,83,84] but not for likes (SMD 3.18, 95% CI –0.25 to 6.62; *P*=.07; *I*^2^=98%; GRADE low) [45,83,85]. The largest effects on engagement were evident in social media campaigns established over 3 to 18 months, usually featuring coordinated, paid social media strategies; daily posts; visually appealing formats; topical hashtags; and @mentions targeting relevant organizations, government resources, and events [53,63,83-85]. Large effects on engagement were also observed in studies featuring a series of live journal clubs [75,78]. Smaller effects of social media on engagement were reported by 1 RCT [45] that used a 14-day Twitter campaign (Figure 3 [45,53,63,75,78,83-85]).

#### Between-Group Effects of Different Platforms, Formats, and Strategies on Engagement

Pooled findings indicated a large between-group effect on engagement in favor of Twitter (vs Facebook, Instagram, and LinkedIn; SMD 1.15, 95% CI 0.21-2.10; *P*=.02; *I*^2^=79%; GRADE low; Multimedia Appendix 3) [47,67,68].

Pooled findings showed large between-group effects on engagement in favor of posts with images compared to no images. Effects were significant for interactions (SMD 1.24, 95% CI 0.53-1.96; *P*<.001; *I*^2^=98%; GRADE low) but not for likes (SMD 0.87, 95% CI –0.40 to 2.14; *P*=.18; *I*^2^=88%; GRADE low; Multimedia Appendix 3) [41,45,49,52,56,72].

Pooled findings indicated a small effect on post interactions of social media strategies with participation by influential others (including patients, authors, and non–peer-reviewed news sources; SMD 0.26, 95% CI 0.13-0.39; *P*<.001; *I*^2^=82%; GRADE low) [54,71,73]. Evidence from individual studies also showed large effects of social media influencers with >1000 followers [52], morning and weekday posting [72], and hashtags [72] and a small effect of @mentions [52] (Multimedia Appendix 3).

### Direct Dissemination

In total, 23 studies (including 8 RCTs [39-45,48]) evaluated direct dissemination (10 in link clicks [41,45,49,51,53,56,73,79,80,83], 13 studies reported article views [39,40,42-44,48,63,69,70,74,81,84,87], 8 measured PDF downloads [40,44,48,50,63,69,79,81], and 9 assessed the altmetric score [44,45,51,69,74,77,79,83,87]).

#### Effects of Social Media Compared to No Social Media on Direct Dissemination

**Figure 4:**
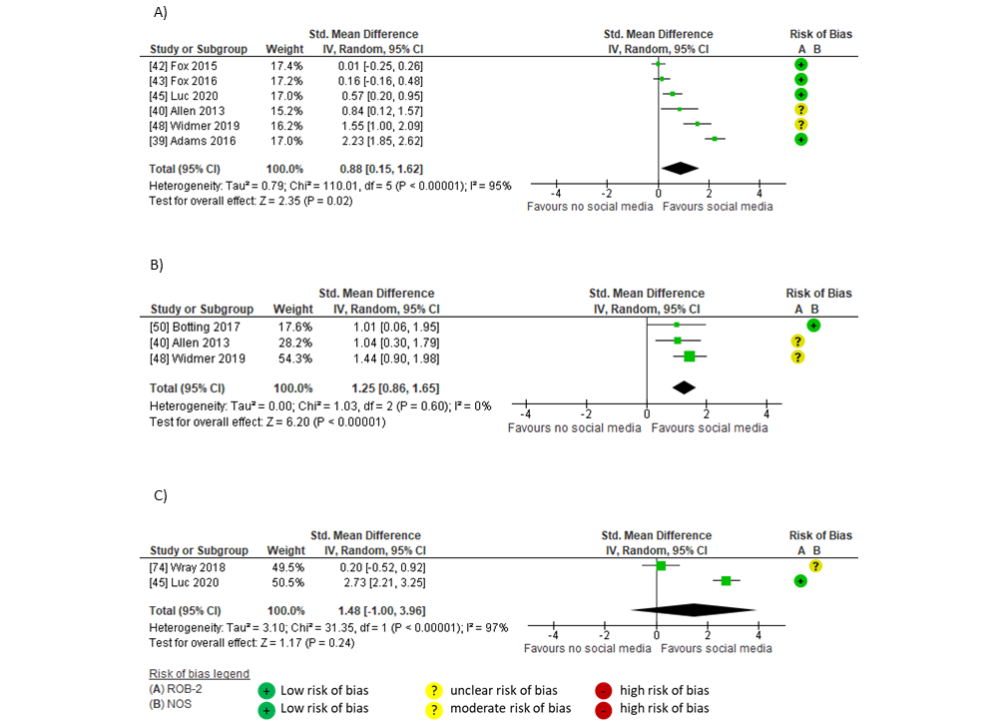
Meta-analyses of between-group effects of social media compared to no social media on direct dissemination in (A) link clicks and article views, (B) article downloads, and (C) the altmetric attention score.

Pooled data showed large between-group effects of social media on direct dissemination. Effects were significant for link clicks or article views (SMD 0.88, 95% CI 0.15-1.62; *P*=.02; *I*^2^=95%; GRADE moderate) [39,40,42,43,45,48] and article downloads (SMD 1.25, 95% CI 0.86-1.65; *P*<.001; *I*^2^=0%; GRADE high) [40,48,50] but not for the altmetric attention score [45,74] (SMD 1.48, 95% CI –1.00 to 3.96; *P*=.24; *I*^2^=97%; GRADE low; Figure 4 [39,40,42,43,45,48,50,74]). Studies that reported the largest effects of social media on direct dissemination used campaigns including professional social media marketing and scheduling tools (Social Bro, Hootsuite, Sprinkler, and Spredfast); multiple posts per day; multiple platforms (Twitter, Weibo, Facebook, and LinkedIn); or multiple accounts on 1 platform, link, and blog [39,40,45,48,50]. The studies that showed the smallest effects of social media on direct dissemination used social media campaigns that posted less than once a day (approximately 0.36 [42] and 0.30 [43] posts per day or once a month publicizing a Twitter journal club [74]).

#### Within-Group Effects of Social Media on Direct Dissemination

**Figure 5:**
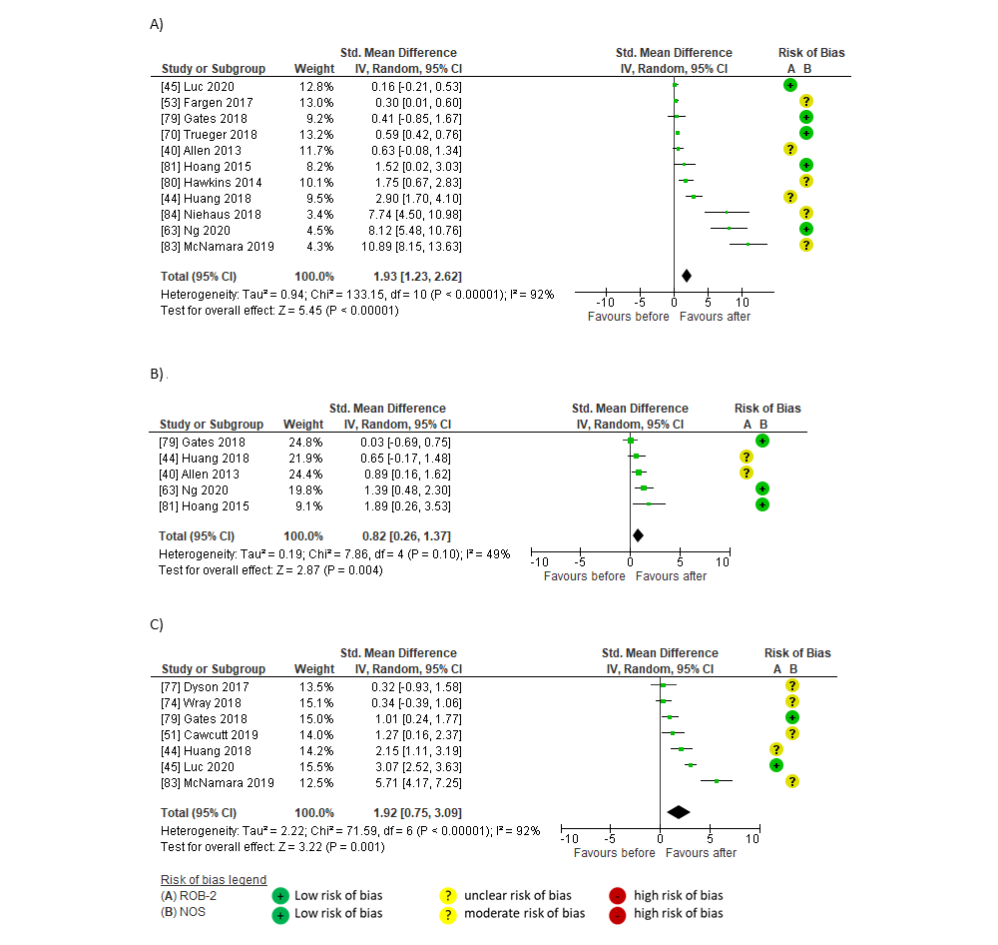
Meta-analyses of within-group effects after social media interventions on direct dissemination in (A) link clicks or article views, (B) article downloads, and (C) the altmetric attention score.

Pooled findings indicated a large, significant within-group effect on direct dissemination after social media interventions compared to before in link clicks or views (SMD 1.93, 95% CI 1.23-2.62; *P*<.001; *I*^2^=92%; GRADE high) [40,44,45,53,63,70,79-81,83,84], article downloads (SMD 0.82, 95% CI 0.26-1.37; *P*=.004; *I*^2^=49%; GRADE moderate) [40,44,63,79,81], and altmetric attention score (SMD 1.92, 95% CI 0.75-3.09; *P*=.001; *I*^2^=92%; GRADE moderate) [44,45,51,74,77,79,83]. Studies that reported the largest effects used campaigns that included coordinated or paid social media software [63,83,84], posting at least once a day [45,63,83,84], visually attractive elements and links [44,45,63,83,84], hashtags, @mentions [45,83,84], multiple platforms (including Twitter and Facebook) [40,44,63,80,81], multiple accounts [40,44,45], associated blogs [40,44,45,79,81], podcasts [81], and tweet chats [51,80] (Figure 5 [40,44,45,51,53,63,70,74,77,79-81, 83,84]).

#### Between-Group Effects of Different Platforms, Formats, and Strategies on Direct Dissemination

Pooled findings showed no significant effect of the platform on direct dissemination (SMD 0.92, 95% CI –1.21 to 3.04; *P*=.40; *I*^2^=99%; GRADE low); both Twitter and Facebook appeared effective [48,70] (Multimedia Appendix 3).

Pooled findings indicated large effects in favor of posts with images, particularly infographics, compared to no images. Effects were significant for link clicks or article views (SMD 1.18, 95% CI 0.27-2.10; *P*=.01; *I*^2^=88%; GRADE low) [41,44,45,56,69] and for altmetric attention score (SMD 1.19, 95% CI 0.04-2.35; *P*=.04; *I*^2^=83%; GRADE low) [44,45,69] but not for article downloads (SMD 0.26, 95% CI –0.32 to 0.83; *P*=.38; *I*^2^=0%; GRADE low) [44,69]. Evidence from individual studies also highlighted large, significant effects of podcasts compared to infographics and standard posts on direct dissemination [69] and a positive effect on link clicks of posts with links compared to posts with infographics [49] (Multimedia Appendix 3).

There were insufficient studies comparing the effect of social media strategies on direct dissemination for pooled data analyses. Evidence from individual studies showed a large, significant effect on link clicks in favor of posting on Tuesdays, Wednesdays, and Saturdays compared to the other days of the week [53] and no effects of time of year [42,43] or non–peer-reviewed news source involvement [73] (Multimedia Appendix 3).

### Impact

A total of 13 studies (including 3 RCTs [46-48]) evaluated impact (7 assessed article citations [46,48,54,55,58,59,65], 3 investigated impact factors [54,61,64], and 4 examined changes in thinking or behavior [47,57,60,82]).

#### Effects of Social Media Compared to No Social Media on Impact

**Figure 6:**
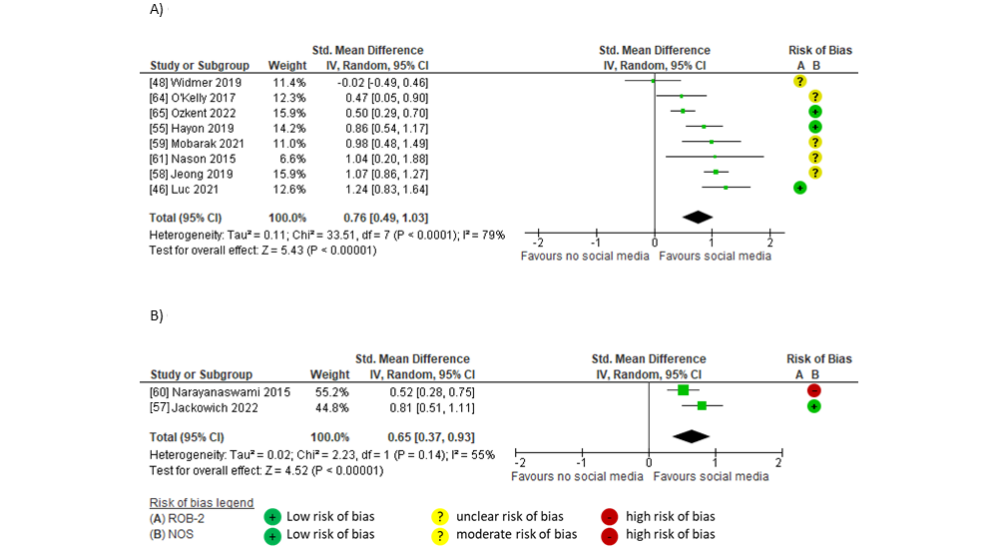
Meta-analysis of the between-group effect of social media interventions compared to no social media interventions on impact in (A) citations and (B) thinking and practice.

Pooled findings indicated a moderate between-group effect of social media compared to no social media on citations (SMD 0.76, 95% CI 0.49-1.03; *P*<.001; *I*^2^=79%; GRADE moderate) [46,48,55,58,59,61,64,65] and thinking and practice (SMD 0.65, 95% CI 0.37-0.93; *P*<.001; *I*^2^=55%; GRADE low) [57,60]. The largest effects on impact were shown in studies that used social media interventions, including links and relevant @mentions [46,55,57-61] in campaigns that often had relatively short durations (14 days [46] to ≤12 months [57-61]). Larger effects on citations were shown in studies sharing articles on broad topics (such as urology or surgery) [46,55,58,59,61], whereas effects on knowledge and practice were evident in social media campaigns that were focused on a specialist topic (eg, persistent genital arousal disorder [57] and complementary and alternative medicine in multiple sclerosis [60]). Studies that showed smaller between-group effects of social media on impact either described social media intervention with infrequent posts on social media (7 times per month, approximately 0.3 posts per day) [48] or without indicating post frequency [64,65] (Figure 6 [46,48,55,57-61,64,65]).

#### Within-Group Effects of Social Media on Impact

**Figure 7:**
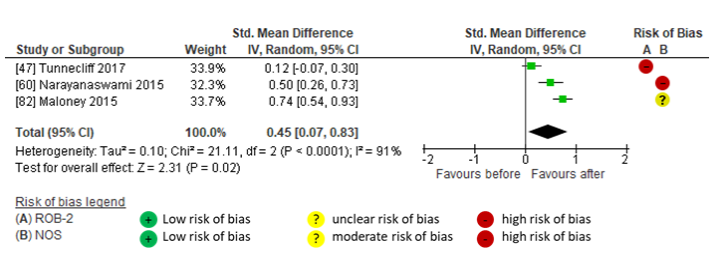
Meta-analysis of within-group effect after social media interventions on thinking and practice.

There were insufficient studies comparing the impact on citations after social media interventions compared to before; however, pooled findings showed a small within-group effect on thinking and practice after social media interventions (SMD 0.45, 95% CI 0.07-0.83; *P*=.02; *I*^2^=91%; GRADE low) [47,60,82]. One RCT [47] and 1 nonrandomized study [82], both concerning tendinopathy practice points with links to research articles or evidence-based podcasts shared for 2 weeks on Twitter and Facebook, reported differing effect sizes on thinking and practice (small and nonsignificant in the RCT [47] but moderate and significant in the nonrandomized study [82]). Another nonrandomized study [60] also reported a moderate effect of a paid targeted social media advertising campaign on Twitter, Facebook, LinkedIn, and YouTube that included article links, images, videos, podcasts, and a live tweet chat with a prominent organization that impacted knowledge, attitudes, and behavior regarding complementary and alternative medicine in multiple sclerosis (Figure 7 [47,60,82]).

#### Between-Group Effects of Different Platforms, Formats, and Strategies on Impact

There were insufficient studies comparing the effect of social media platforms on impact for pooled data analyses. Evidence from individual studies showed a large, significant effect on the journal impact factor of posting on ≥3 social media platforms [64]; a positive association between the journal impact factor and the number of social media platforms used [59]; and a small, nonsignificant effect of platform in favor of Twitter (vs Facebook) on knowledge and practice changes [47] (Multimedia Appendix 3).

No included studies compared the effect of different formats on impact.

Pooled findings indicated a large, nonsignificant effect on citations of author tweets in addition to standard journal social media strategies (SMD 1.00, 95% CI –0.84 to 2.84; *P*=.29; *I*^2^=98%; GRADE very low; Multimedia Appendix 3) [54,55].

### Certainty

Considering all the evidence, there was high certainty that social media is effective for the dissemination of research to health care practitioners. The outcome with the highest certainty across all comparisons was direct dissemination. Evidence was insufficient to determine the size and certainty of between-group effects of social media compared to no social media on reach and engagement; however, there was moderate certainty of large and moderate effects of social media interventions on direct dissemination and impact, respectively. After social media exposure, there was moderate certainty of large, positive within-group effects on reach, engagement, and direct dissemination, whereas there was low certainty of a small effect on impact. Certainty was generally low regarding the size of the effects of platforms, formats, and strategies on each outcome. However, the direction of effects was consistently in favor of using multiple platforms (particularly Twitter and Facebook), using images (particularly infographics), and involving influential others in social media campaigns.

The level of certainty about the size of the effects in favor of social media was different depending on the outcome of interest and study characteristics. There was a tendency for smaller effect sizes in RCTs and studies with less-intensive social media interventions. The certainty ratings were lowered for all comparisons due to the variability of the included study designs, many of which were descriptive and not designed for rigorous quantitative evaluations, meaning most included studies had moderate risk of bias. Consistency and precision of effect size estimates were also threatened by the low number and heterogeneity of studies included in some comparisons. However, for some comparisons, certainty was uprated due to the large magnitude of estimated effects and potential dose-response gradients between the intensity of social media interventions and their effectiveness.

## Discussion

### Principal Findings

There was evidence of the effectiveness of social media for the dissemination of research evidence to health care practitioners. All the included studies reported some findings in favor of social media, although there was considerable heterogeneity in effect sizes, and study quality was mixed. Effect sizes of social media effectiveness were influenced by the frequency, intensity, and composition of social media interventions. Effectiveness was enhanced by the use of multiple social media platforms (including Twitter and Facebook); multiple social media accounts; ≥1 social media post per day; appealing formats (including infographics or other visual media, blogs, and links to articles); professional social media marketing and scheduling tools and involving relevant and influential people, organizations, and events in social media campaigns.

Our findings that social media was beneficial for the dissemination of research evidence to practitioners concurred with existing literature about the largely positive impact of social media on dissemination [10,21-23,28,65,90-93]. Quantitative analyses in other studies have revealed positive correlations between social media use and the dissemination and impact of health research evidence [65,91-93]. Narrative and qualitative reviews have highlighted benefits of social media for clinicians including connectedness and network accessibility to all (particularly with increasing use of communication technology and mobile apps in practice and day-to-day life), the large audience of practitioners and policy makers that uses social media (particularly for information acquisition and educational purposes), and the effectiveness of social media to deliver clinical guidelines and research evidence–based information that could be implemented in practice [21-23,28,90]. Reviews also highlighted the challenges of synthesizing social media effectiveness for the dissemination of information due to the heterogeneity of how social media are studied and used [22,28,90].

Social media campaigns to disseminate research evidence to health care practitioners should consider target outcomes because specific features enhanced the 4 outcomes of interest differently (Table 1).

Our quantitative findings in favor of using multiple social media platforms, including Twitter (for engagement in particular) and Facebook, concurred with existing literature that recommends using a range of social media platforms [23,28,90,94] and highlights the prominence of Twitter for research dissemination [28] and Twitter and Facebook for e-professionalism [22]. New knowledge from our systematic review includes the potentially beneficial effect of using multiple social media accounts on the same platform. This may also link with the apparent dose-response relationship between effectiveness and the average number of social media posts per day.

There was consistent evidence to support the use of visual media, particularly infographics for better reach, engagement, and direct dissemination; our findings suggest that social media effectiveness may also be enhanced by other post formats including podcasts, blogs, questions, and *practice points* to disseminate research evidence on social media. This adds to previous literature, which recommended posting a range of appealing multimedia that is accessible, useful, relevant, authentic, and credible [10,23,28,90]. Our review also highlighted the importance of including links to original research; comprehensive infographics or practice points posted on social media might reduce the likelihood of viewing the original article by link click [49]. Nevertheless, including links offers the viewer the opportunity to check the authenticity and credibility of the information in a post. Including links may also facilitate the delivery of simple, clear, and practice-relevant messages without scientific language [88] because the main message from the research can be easily understood and accessible to all practitioners, while further details can be sought by accessing the link.

Our findings corroborate existing recommendations to identify and involve key influencers, organizations, events, communities, #hashtags, and @mentions and to use professional tools to plan sustained, scheduled, and regular posts to overcome the transient nature of social media [10,23,28,90]. Our systematic review extends existing recommendations by identifying that posting at least once a day on average (often achieved using multiple accounts, platforms or both) was more effective than less-intensive social media strategies, suggesting a dose-response relationship between post frequency and social media effectiveness. This dose-response relationship may explain the negligible effect of one journal’s social media campaign tested in 2 RCTs included in this systematic review [42,43].

The optimal timing of social media posts and campaigns for the dissemination of research evidence to practitioners is contentious in existing literature [53,95,96]. Optimal timing may be outcome dependent; in this review, different times of the day and different days of the week were more effective depending on the outcome measured (Table 1). Similarly, while reach and engagement may be enhanced by established social media initiatives extending over months or years, impact may be best achieved in shorter, targeted social media campaigns over days or weeks.

Our findings suggested a tendency for greater impact on thinking and practice from social media featuring targeted topics or specialist areas of health care. This concurred with previous reviews that found that the dissemination of clinical guidelines or clear, evidence-based behavior change messages, such as *practice points*, may increase impact with health care practitioners [90,94]. This also resonates with recommendations in the existing literature to consider the target audience when selecting platforms, formats, and strategies that optimize content for the dissemination of research evidence [23,88,90,94]. For instance, the choice of platform and content about pediatric colorectal cancer should be guided by the understanding that, in this field, Twitter is typically used to share research evidence, Facebook is used for support offered by nonprofit organizations, and Instagram is used for sharing personal stories [67].

Our findings suggested a tendency that social media was more effective on impact (in citations) when used to share research articles about broad topics. Thus, there may be interactions between the content of research evidence posts and the effectiveness of dissemination on social media, regardless of platform, format, or strategy. Indeed, there are indications in the wider literature that research evidence source, topic, and post content may influence how effectively it can be disseminated on social media. For example, geographically, compared to the United States, authors from Europe, and UK clinical guidelines, achieve better altmetric attention scores and citation rates [58,97]. Published articles may achieve greater reach, research conference posts may receive greater engagement [73], and clinical guidelines may better influence thinking and practice [90]. Research evidence that aligns with “hot topics” on social media may also achieve better reach and engagement [68,98]. Engagement, direct dissemination, and citation rates may be better for open-access articles (especially reviews) that are recently published with shorter titles (which are provocative, interrogative, or declarative and free from methodological description), with a greater number of authors, and by higher impact factor journals (often with a larger social media presence in terms of followers and number of tweets per month) [54,58,59,65,99-101]. Depending on the health topic, posts with humor, shock value, inaccuracies, rumors, or emotional content might achieve better reach [66,102,103]; posts with practical guidance may be more likely to be shared [66,102,104,105]; and short videos with positive titles might receive more likes and comments [104].

**Table 1:** Summary of the social media characteristics that may enhance effectiveness depending on the outcome.

Outcomes	Reach	Engagement	Direct dissemination	Impact
Platforms	Multiple platforms (including Twitter and Facebook)	Twitter	Multiple platforms (including Twitter and Facebook)	≥3 platforms
Format	Images (particularly infographics) Blogs Microblogs	Images (particularly infographics) Appealing media (eg, videos, enticing statements, or questions)	Images (particularly infographics) Podcasts Blogs Links to articles	Clinically relevant and useable posts Microblogs Links
Strategies	Involvement of influencers and organizations	Involvement of influencers, patients, authors, and organizations Relevant @mentions and hashtags	Intensive strategies Relevant @mentions and hashtags	Targeting specific audiences or specialism for practice Broader topics for citations Relevant @mentions
Timing	≥1 posts per day Established campaigns sustained over months or years	Scheduled posts (eg, weekday mornings) Established campaigns coinciding with targeted events and government resources	≥1 posts per day Any time of the year Scheduled posts (eg, on Tuesdays, Wednesdays, and Saturdays)	Frequent posts Brief but focused campaigns (eg, a year or less)
Resources	Multiple social media accounts Social media marketing tools	Regular established live events (eg, journal clubs and tweet chats) Paid social media advertising	Multiple social media accounts Social media marketing and scheduling tools	Paid social media advertising Live events involving prominent organizations

### Strengths and Limitations

The key strengths of this systematic review are the quantitative meta-analytical methods used that allow robust conclusions based on cumulative evidence about the effective use of social media for the dissemination of research evidence to practitioners. Methodological limitations have been discussed in the published protocol [29]. The search strategy was comprehensive, using multiple reviewers to ensure reliability and comprising a range of study designs including RCTs. However, no studies were identified investigating the effectiveness of social media for the dissemination of research evidence to social care practitioners. There were also relatively few RCTs, and the mixed quality of the included studies reduced the certainty of evidence about effect sizes for some outcomes. A wide range of social media interventions and research evidence content were represented by the included studies; this reduced the risk of confounding by topic, source, or content. However, time may have reduced the consistency of findings between included studies because the research and social media landscapes are rapidly evolving and have changed significantly from 2010 to 2023 [11,14]. Furthermore, the time frame of outcome measurement can influence potentially transient social media effectiveness [55]. Effectiveness was evaluated thoroughly in 4 outcome domains, comprising 9 outcomes of interest and multiple measures; this helped to accommodate the variability of reporting and design in the included studies. Although the diversity of the included studies threatened the consistency of effect sizes, the direction of effect in favor of social media was consistent. The heterogeneity of findings was ameliorated using a random effects model for more conservative estimates of effect size than a fixed effects model. Using 4 outcome domains added nuance to the existing understanding and facilitated the development of clear suggestions about how to optimize social media effectiveness for the dissemination of research evidence to health care practitioners. However, a cautious interpretation of a causal relationship between dissemination effectiveness and specific social media tactics is required. Effects may have been inflated by other confounders; for example, larger organizations with greater resources for public relations not only can post more frequently on social media but also may have greater reputational influence and share higher-quality research.

### Conclusions

In conclusion, social media were effective for disseminating research evidence to health care practitioners. Large effects of and after social media interventions on measures of direct dissemination were particularly evident. There may be a dose-response relationship between the intensity of the social media campaign and its effectiveness. Selected social media intervention characteristics including platforms, formats, and strategies may enhance reach, engagement, direct dissemination, and impact of research evidence for practitioners. Future research directions include repetition of this review to keep up with the rapidly evolving use of social media for research dissemination; quantitative testing of the potential dose-response relationship between dissemination effectiveness and social media frequency and intensity; and further evaluation and exploration of how different practitioner groups, particularly social care practitioners, use social media to access research evidence.

## Appendix

Multimedia Appendix 1 PRISMA (Preferred Reporting Items for Systematic Reviews and Meta-Analyses) checklist.

Multimedia Appendix 2 Search strategy.

Multimedia Appendix 3 Description of the included articles and full details of meta-analyses.

## Abbreviations

GRADE: Grading of Recommendations, Assessment, Development, and Evaluations
PRISMA: Preferred Reporting Items for Systematic Reviews and Meta-Analyses
RCT: randomized controlled trial
SMD: standardized mean difference

## Data Availability

The data sets generated and analyzed during this study will be available from the corresponding author on reasonable request. Findings from this systematic review will be disseminated at academic conferences and promoted using a range of social media strategies.
