# Supplementary material for: Examining the Effectiveness of Social Media for the Dissemination of Research Evidence for Health and Social Care Practitioners: Systematic Review and Meta-Analysis
Source: J Med Internet Res. 2024 Jun 5;26:e51418. doi: 10.2196/51418 (PMC11187521; doi:10.2196/51418)
Supplement: Multimedia Appendix 3 [file jmir_v26i1e51418_app3.docx]

# Multimedia Appendix 3

| Index | Page Number |
| --- | --- |
| Description of included studies (Table A) | 2 |
| Quantitative comparisons of effect sizes for each outcome of interest (Table B) | 14 |
| Reach additional results and figures | 16 |
| Engagement additional results and figures | 19 |
| Direct dissemination additional results and figures | 22 |
| Impact additional results and figures | 25 |
| References of included studies | 26 |

## Included studies

Fifty articles published between 2013 and 2022 were included; nine were randomised controlled trials (RCTs) [1-10], 27 were non-randomised designs [11-36], 22 were before after designs or additional comparisons [2, 6, 7, 9, 13, 15, 22, 25, 32, 36-48], and three case studies [49-51] (see Table A).

Table A: Characteristics of included studies

| **Article /**  **Risk of Bias** | **Subjects**  **(specialism)** | **Platform**  **(date)** | **Format** | **Strategy** | **Design (comparison)** | **Outcomes** |
| --- | --- | --- | --- | --- | --- | --- |
| Adams et al 2016 [1]  ☺ Low risk of bias | 170 Articles (Cochrane systematic reviews about  Schizophrenia) | Twitter,  Weibo  (23 weeks, 1 July 2014 to 13 January 2015) | Text post (including article title, extract from abstract, results or discussion, a question, or pithy statement related to the evidence) | Posts released on the SM accounts of the Cochrane Schizophrenia group, posts were Hootsuite scheduled, Social Bro web tool guided, 3 posts per day at 10:30, 13:00 and 15:00 GMT, in English on Twitter then 8 hours later in Chinese on Weibo. | RCT  (SM vs No SM) | Dissemination: Article views after 7 days showed a large effect in favour of SM |
| Allen et al 2013 [2]  😐 unclear risk of bias | 16 Blogs  (Featuring peer-reviewed articles of interest to the blog audience about Pain) | Twitter, Facebook, LinkedIn,  (6 months, 14 August 2011 to 2 February 2012) | Text post with link to Blog (bodyinmind.org) that included link to original article. | Posts released on multiple accounts linked to the Body and Mind blog on a Tuesday (between 6 and 7 am) or between 11 pm Thursday and 2 am Friday, Australian Eastern Summertime. | RCT  (SM vs No SM, Before vs After) | Dissemination: Article views and Article downloads after 7 days showed moderate and large effects in favour of SM and increases after SM exposure |
| Chapman et al 2019 [3]  ☺ Low risk of bias | 41 Articles  (British Journal of Surgery systematic reviews and empirical research  about Surgery) | Twitter  (4 months between August and December 2018) | Text post (standard abstract with screen shot image) or plain English abstract, or infographic (all with links to original article) | Post released on a clinical academic journal's SM @BJSurgery. Each article tweeted once, then again after 14 days | RCT  (Format vs Format) | Reach: Practitioner number and Impressions after 2 weeks showed a moderate and large effect respectively in favour of infographics  Engagement: Total Interactions after 2 weeks showed a moderate non-significant effect in favour of infographics  Dissemination: Link clicks after 2 weeks showed a small effect in favour of infographics |
| **Article /**  **Risk of Bias** | **Subjects**  **(specialism)** | **Platform**  **(date)** | **Format** | **Strategy** | **Design (comparison)** | **Outcomes** |
| Fox et al 2015 [4]  ☺ Low risk of bias | 243 Articles (Circulation journal articles about Cardiology and cardiovascular medicine) | Twitter, Facebook  (11 months (September 10, 2013 to August 12, 2014) | Text post (with links and figure images where possible) | Posts released on a clinical academic journal’s SM (@CircAHA). Each article posted once on Monday to Thursday, on the formal publication of each article. | RCT  (SM vs No SM) | Dissemination: Article views after 30 days showed a non-significant effect in favour of SM |
| Fox et al 2016 [5]  ☺ Low risk of bias | 152 Articles  (Circulation journal articles about Cardiology and cardiovascular medicine) | Twitter, Facebook  (8 months January 13, 2015 to September 22, 2015) | Text post (with links and figure images where possible) | Posts released on a clinical academic journal’s SM (@CircAHA) using a paid targeted SM strategy and 2-7 general posts per day. Articles posted contemporaneous with the formal publication date and reposted the next day at 11AM and 3PM up to a total of 3 posts per article. | RCT  (SM vs No SM) | Dissemination: Article views after 30 days showed a non-significant effect in favour of SM |
| Huang et al 2018 [6]  😐 unclear risk of bias | 24 Articles  (Canadian Journal of Emergency Medicine (CJEM) original research articles about emergency medicine) | Twitter, Facebook  (12 months (July 2016 and June 2017) | Text post (with link and image (article screenshot OR infographic), plus blog post including infographic) | Posts released on a clinical academic journal’s SM (CJEM and CanadiEM accounts x 4, plus blog post on the CanadiEM website). Articles promoted within 1-2 weeks of the official publication date (all articles previously published online). | RCT  (Format vs Format, Before vs After) | Dissemination: Article Abstract Views, full text article downloads, Altmetric attention score after 30 days showed large effects in favour of infographics and large increases after SM exposure. |
| Luc et al 2020 [7]  ☺ Low risk of bias | 112 Articles (The Annals of Thoracic Surgery and The Journal of Thoracic and Cardiovascular Surgery original research articles about cardiothoracic surgery) | Twitter  (14 days from June 4 to June 17, 2018) | Text post (with link, photo and @involved authors and institutions) | Posts released on a personal Twitter account belonging to a TSSMN delegate, each tweet was retweeted by each of 11 other TSSMN delegates and the official @TSSMN handle. Articles promoted 4 per day for 14 days. | RCT  (SM vs No SM,  Format vs Format,  Before vs After) | Reach: Physicians and Impressions showed a large effect in favour of SM  Engagement: Likes and Total interactions (including retweets, hashtag clicks, detail expansion, media engagements) showed small non-significant effects in favour of SM  Dissemination: Link clicks and Altmetric attention score and after 7 days showed large and moderate effects in favour of SM and small effects in favour of images |
| **Article /**  **Risk of Bias** | **Subjects**  **(specialism)** | **Platform**  **(date)** | **Format** | **Strategy** | **Design (comparison)** | **Outcomes** |
| Luc et al 2021 [8] (☺ as above) | Follow up study, subjects as above | Follow up study, platform as above | Follow up study, format as above | Follow up study, strategy as above | RCT  (SM vs No SM) | Impact: citations after 1 year showed a large effect in favour of SM |
| Tunnecliff et al 2017 [9]  ☹ high risk of bias | 8 Microblogs (featuring practice points based on peer-reviewed journal articles or podcasts about Tendinopathy) | Twitter, Facebook  (2 weeks between August to October 2014) | Text post (up to 140-character practice point) with link to article or podcast. On Facebook, 2–6 short sentences of key concepts as well | Post released evenly over a 2-week period at the same times on each platform to separate groups of followers on Twitter (n=140) and Facebook (n=177) | RCT  (Platform vs Platform, Before vs After) | Engagement: Likes per post and shares per post after 2 weeks showed a large effect in favour of Twitter  Impact: Knowledge, use of research in practice, change in practice after 2 weeks showed a small effect in favour of Twitter |
| Widmer et al 2019 [10]  😐 unclear risk of bias | 68 Articles (Mayo Clinic Proceedings journal original research articles and editors’ letters about general internal medicine) | Twitter, Facebook, Linked In  (3 months October 2015 to January 2016) | Text posts (with links and images where available) | Posts released on multiple SM accounts associated with the journal (@ElsevierConnect, @MayoProceedings, @MayoClinic), 7 posts per month (4 Twitter, 2 Facebook and 1 Linked In) distributed evenly throughout the month at different times of day | RCT  (SM vs No SM) | Dissemination: Article views and article downloads after a month showed a large effect in favour of SM and Twitter  Impact: Citations after 1-2 years showed a non-significant effect of SM |
| Barlow et al 2020 [11]  ☺ Low risk of bias | 40 Tweets (within @theABofPharmaC microblog featuring peer reviewed research based clinical pearls  about Pharmacy) | Twitter  (10 months 1 January 2019 to 11 November 2019) | Standard text Tweet (with link) with or without infographics | Posts released on one Twitter account (frequency not reported) | Retrospective non-randomised comparison (Format vs Format) | Reach: Impressions after an unspecified time showed a large effect in favour of infographics  Engagement: Likes and Total interactions (including retweets, replies and other interactions) showed a large effect in favour of infographics  Dissemination: Link clicks showed a non-significant effect in favour of posts with links not infographics |
| Bolderston et al 2022 [37]  😐 unclear risk of bias | 12 Articles per year (featured in Tweet Chat journal clubs about Radiography) | Twitter  (March 2015 to February 2020) | Format of Tweets not stated, Live Tweet chat and Blog for each article included in the journal club | Posts released live from one Twitter account for an hour every month | Observational over 5 years (Before vs After SM) | Reach: Impressions and practitioner participants after each monthly journal club showed a large increase after the SM campaign  Engagement: Interactions with blog after each month showed a large increase after the SM campaign |
| **Article /**  **Risk of Bias** | **Subjects**  **(specialism)** | **Platform**  **(date)** | **Format** | **Strategy** | **Design (comparison)** | **Outcomes** |
| Botting et al 2017 [12]  ☺ Low risk of bias | 30 Articles (written by academics in the Division of Language and Communication Sciences, City University London) | Twitter  (2 years, 2012-2013) | Standard text Tweet with link to Blog | Posts released on 2 student, staff and official university Twitter accounts, tweeted 3 times per day for first 3 days and then once a week for the remainder of the month. Blog released monthly for 3 consecutive months per article. | Retrospective non-randomised comparison  (SM vs No SM) | Dissemination: Article downloads after 4 months showed a large effect in favour of SM |
| Buckarma et al 2017 [49]  ☹ high risk of bias | 1 Article (peer-reviewed "Surgical never events and contributing human factors" and article  Blog about surgery) | Twitter, Instagram, Linked In, Spotify (podcasts), YouTube and TikTok  (10 months, May to October 2015) | Format of posts unspecified links to 650-word Blog written by authors | Posts released by Kevin MD (a blog with over 2 million monthly page views, and associated with multiple SM accounts) | Case study with prediction model control  (SM vs No SM,  Before vs After) | Reach: Views after monthly data stabilised for 2 months favoured SM  Engagement: shares on Twitter and Facebook after monthly data stabilised for 2 months favoured SM  Dissemination: Article views and Altmetric attention score after monthly data stabilised for 2 months favoured SM |
| Carley et al 2018 [38]  😐 unclear risk of bias | 510 Articles (published on St Emlyn's blog over 4.5 years  about emergency medicine) | Twitter, Facebook, Instagram  (4.5 years, June 2012 to January 2017) | Standard text Tweets with links to Blog on WorldPress and podcast | Posts released in association with a blog by an editorial team (2 lead editors and over time a multi-professional editorial team of 12 clinicians from the USA, UK and Australia) timing not stated. | Observational case study over 4.5 years  (Before vs After) | Reach: views of blog each month showed a large increase after SM exposure |
| Cawcutt et al 2019 [13]  😐 unclear risk of bias | 8 Articles (included in a one-hour Physician’s Weekly Tweet chat about workforce gender equity) | Twitter  (1 hour, April 10, 2018) | Text and Image based Tweets with questions and links to articles | Posts released in one off live Tweet chat hour by Physician's Weekly Twitter account, questions were designed by the guest host and study author in advance of the event. One influencer account compared to nine standard accounts. | Non-randomised comparison (Strategy vs Strategy,  Before vs After) | Reach: Impressions after 2 weeks showed a large effect in favour of influencer reach  Altmetric Attention Score after 49 days, showed a large increase after SM exposure |
| **Article /**  **Risk of Bias** | **Subjects**  **(specialism)** | **Platform**  **(date)** | **Format** | **Strategy** | **Design (comparison)** | **Outcomes** |
| Cevik et al 2019 [14]  😐 unclear risk of bias | Conference Hashtag (on posts at the 2017 and 2018 European Congress of Clinical Microbiology and Infectious Diseases) | Twitter  (2 years of two 4-day conferences, April 2017 and April 2018) | Text Tweets, some with links, media (video or image), @ mentions, # additional hashtags | Posts released during each conference, comparison between regular user accounts or influencer accounts (with >1000 followers) | Retrospective observational study over 2 years (Format vs Format,  Strategy vs Strategy) | Engagement: Retweets (shares) each month showed a large effect in favour of influencer accounts and small effects in favour of media (videos or images), @mentions and # hashtags |
| Dyson et al 2017 [39]  😐 unclear risk of bias | 5 Articles per month (featured in an evidence-Based Child Health: A Cochrane Review Journal blog) | Twitter, Facebook (22 weeks, November 2014 to April 2015) | Text posts with links, @ mentions and # hashtags, Blog posts and live Tweet chats | Posts released 21 times per week from the Cochrane Child Health Twitter and Facebook accounts, linked with weekly blog (WorldPress) and live monthly journal club (Tweet chat). | Observational study over 22 weeks  (Before vs After) | Dissemination: Altmetric Attention Score after each month showed a non-significant increase after SM exposure |
| Fargen et al 2017 [15]  😐 unclear risk of bias | 191 Articles (published by the  Journal of Neuro-Interventional Surgery (JNIS)) | Twitter, Facebook  (9 months, February 2015 to December 2015) | Text posts with links, images of figures and @ first author | Posts release within a few days of new article release Journal of Neuro-Interventional Surgery (JNIS) official and assistant editor Twitter accounts and Society of Neuro-Interventional Surgery (SNIS) and JNIS Facebook pages, with author involvement in posts | Observational over 9 months  (Before vs After,  Strategy vs Strategy) | Reach: Impressions each month showed a small increase after SM exposure Engagement: Total interactions each month showed a large increase after SM  Dissemination: Link clicks each month showed a small increase after SM exposure and a large effect in favour of posts on Tuesday, Wednesday and Saturday. |
| Gardhouse et al 2017 [40]  😐 unclear risk of bias | 12 Articles (featured in a monthly journal club Tweet chat about geriatric medicine) | Twitter  (12 months, August 2014 to August 2015) | Text Tweets with questions and a Webcast video of the journal club | Posts released over 24 hours, first hour live webcast video of the journal club, then 23 hours asynchronous Tweet chat using a single account (@GeriMedJC) that announced articles in advance of the live journal club. | Observational over 12 months  (Before vs After) | Reach: Impressions after 24 hours and participants each month showed a large increase after SM exposure  Engagement: Total interactions (tweets, retweets and replies) each month showed a large effect increase SM exposure |
| Gates et al 2018 [41]  ☺ Low risk of bias | 16 Articles (systematic reviews featured in a weekly Cochrane Child health blog) | Twitter  (16-weeks, 5 October 2016 and weekly until 1 February 2017) | Text Tweets with links and images; Blog with plain language summary, 3 key messages in an image, link to full article and citations | Posts released 98 times per week via 4 Twitter accounts: @Cochrane_Child, @TRIPChildHealth @arche4evidence, @TREKKca (Translating Emergency Knowledge for Kids), Blog released once a week. | Observational over 16 weeks  (Before vs After) | Reach: Views of blog and users (practitioners) after 30 days showed a small and large increase respectively after SM  Dissemination: Link clicks and article downloads showed no significant effect, but Altmetric attention score after 30 days showed a large increase after SM exposure |
| **Article /**  **Risk of Bias** | **Subjects**  **(specialism)** | **Platform**  **(date)** | **Format** | **Strategy** | **Design (comparison)** | **Outcomes** |
| Gunaratne et al 2020 [16]  😐 unclear risk of bias | 15,078 Articles (Eighty-four Pulmonary and Critical Care Medicine journals Tweeted at least once) | Twitter  (5.5 years, June 6, 2011, to January 1, 2017) | Tweet format not specified | Posts were cross referenced to check whether article authors had Tweeted or not. | Retrospective non-randomised comparison  (Strategy vs Strategy) | Engagement: Total Twitter mentions after 1 year showed a small effect in favour of author tweeted articles and higher impact factor journals (≥5)  Impact: Impact factor after 1 year showed a small effect in favour of author tweeted articles and higher impact factor journals (≥5) |
| Hawkins et al 2014 [42]  😐 unclear risk of bias | 6 Articles (selected by the editor of the Journal of the American College of Radiology (JACR)) | Twitter, Facebook  (24 months, July 2013-July 2015) | Text posts including line up, topics and questions relating to the forthcoming Tweet chat | Posts preceded a monthly live Tweet chat featuring one or two guest moderators who were experts on the topic and who had prominent SM presences. | Observational over 24 months  (Before vs After) | Dissemination: Link clicks after each month showed a large increase after SM exposure |
| Hayon et al 2019 [17]  ☺ Low risk of bias | 213 Articles (Tweeted or not Tweeted from 7 prominent urology journals) | Twitter  (37 months preceding 2019) | Tweet format not specified | Posts were tweeted by academic journals (European Urology, Journal of Urology, British Journal of Urology International, Prostate Cancer and Prostatic Disease, Journal of Sexual Medicine, Journal of Endourology, Urology) and cross referenced to check whether article authors had also Tweeted or not. | Retrospective non-randomised comparison  (SM vs No SM,  Strategy vs Strategy) | Impact: Citations after 37 months showed large effects in favour of article Tweeting by journals and additional Tweeting by authors |
| Hoang et al 2015 [43]  ☺ Low risk of bias | 3 Articles (by the same author published in peer-reviewed journals and a Blog about radiology) | Twitter, Facebook, Tumblr  (17 months, April 2013 to September 2014) | Text posts with links to blog, article and/or podcast | Posts released on official SM accounts for the American Journal of Neuroradiology (AJNR) blog, fellow journal club podcast, article links also emailed to AJNR subscribers. | Retrospective observational study over 17 months  (Before vs After) | Reach: Views of blog after each month showed a large increase after SM exposure  Dissemination: Article views and article downloads after each month showed large increase after SM exposure |
| **Article /**  **Risk of Bias** | **Subjects**  **(specialism)** | **Platform**  **(date)** | **Format** | **Strategy** | **Design (comparison)** | **Outcomes** |
| Ibrahim et al 2017 [18]  ☺ Low risk of bias | 44 Articles (featured on SM by the Annals of Surgery Journal) | Twitter  (6-months, July 2016 to December 2016) | Text Tweets with links and either article title or visual abstracts | Posts released twice per article, with a 4-week gap between, on the Annals of surgery Twitter account | Cross over comparison study  (Format vs Format, Before vs After) | Reach: Impressions after 4 weeks showed a large effect in favour of visual abstracts and a moderate increase after SM exposure  Engagement: Retweets after 4 weeks showed a large effect in favour of visual abstracts  Dissemination: Link clicks after 4 weeks showed a large effect in favour of visual abstracts |
| Jackowich et al 2022 [19]  ☺ Low risk of bias | 297 Microblog viewers or non-viewers (as part of a knowledge translation campaign about persistent genital arousal disorder (PGAD)) | Twitter, Facebook, Instagram  (12-months, November 2019 to December 2020) | Text posts with facts, article links, and infographics | Posted released, once a week on each platform, using established SM accounts affiliated with Queens University Sexual Health Research Lab (Facebook @sexlab.ca, Twitter @QSexLab, Instagram @sexlab.ca) | Non-randomised comparison  (SM vs No SM,  Platform vs Platform) | Reach: Impressions (between platforms) viewing microblog 17 months showed a large effect in favour of Twitter  Impact: Measures of thinking (knowledge) and practice (confidence) concerning PGAD after viewing microblog showed a large effect in favour of viewing SM campaign |
| Jeong et al 2019 [20]  😐 unclear risk of bias | 404 Articles (published by peer reviewed Coloproctology journals) | Twitter, Facebook, YouTube  (12-months, June 2015 to May 2016) | Post format not specified | Posts released by official journal SM accounts e.g., @AGSJournal, Techniques in Coloproctology, Colorectal Disease, Diseases of the Colon & Rectum accounts and individual influencer accounts e.g., @LeeLindquistMD | Retrospective non-randomised comparison  (SM vs No SM) | Impact: Citations after 2.5 to 3 years showed a large effect in favour of article promotion by journals on Twitter |
| Lindquist et al 2019 [50]  ☹ high risk of bias | 1 Article (published by the Journal of the American Geriatrics Society) | Twitter  (10 days before 2019) | Text post including title, facts, article and screenshot, one # (or including a question, link, infographic, multiple relevant # and @) | Post released either by official journal Twitter account (@AGSJournal) or by lead author Twitter account (@LeeLindquistMD) | Case Study  (Format vs Format) | Reach: Impressions after 4-8 days favoured the infographic  Engagement: Likes and Retweets (shares) after 4-8 days favoured the infographic |
| **Article /**  **Risk of Bias** | **Subjects**  **(specialism)** | **Platform**  **(date)** | **Format** | **Strategy** | **Design (comparison)** | **Outcomes** |
| Maloney et al 2015 [44]  😐 unclear risk of bias | 173 Microblog viewers (of evidence-based practice points about tendinopathy) | Twitter, Facebook  (2 weeks before 2015) | Text Tweet about implications of research for practice with links to original articles and/or podcasts | Posts released 8 times over 2 weeks by the Monash Tendon Research Group SM feeds. | Prospective intervention study  (Before vs After) | Impact: Measures of thinking (knowledge) and practice (self-reported changes in behaviour) concerning tendinopathy after 1 week showed a moderate increase after SM exposure |
| McNamara et al 2019 [45]  😐 unclear risk of bias | 18 months of Articles (featured on SM by the International Journal of Mental Health Nursing (IJMHN)) | Twitter  (36 months, July 2015 to June 2018) | Text Tweets with links, visually attractive element, quote, key points, Twitter handles @ and hashtags # | Posts released twice daily at 07:00 and 19:00 hours, using @IJMHN twitter feed and Hootsuite for Tweet scheduling. | Observational study over 36 months  (Before vs After) | Reach: Impressions each month showed a large increase after SM exposure  Engagement: Likes and Retweets (shares) each month showed large increases after SM exposure  Dissemination: Link clicks and Altmetric attention score each month showed large increases after SM exposure |
| Mobarak et al 2021 [21]  😐 unclear risk of bias | 80 journals (about general surgery) | Twitter  (1 year, 2019 to 2020) | Post format not specified | Strategy not specified | Retrospective non-randomised comparison  (SM vs No SM) | Impact: CiteScore (citations) after 1 year showed a large effect in favour of journals with Twitter profiles |
| Narayana-swami et al 2015 [22]  ☹ high risk of bias | 144 Guideline viewers (of an evidence-based clinical practice guideline about complementary and alternative medicine in multiple sclerosis published in Neurology journal) | Twitter, Facebook, Linked In, YouTube  (7 months, February 2014 to September 2014) | Text posts with links, images, videos, podcasts, and live Tweet chats | Posted released over 90 days on SM advertising to targeted audiences where possible and live 1 hour Tweet chats at 12 noon with target organisations (TIME Magazine, the National Multiple Sclerosis Society (NMSS), and Beth Israel Deaconess Medical Centre in Boston) | Non-randomised comparison (SM vs No SM  Before vs After) | Impact: Measures of thinking (awareness) and practice (behaviour change) after each month showed a moderate effect in favour of SM and after SM |
| **Article /**  **Risk of Bias** | **Subjects**  **(specialism)** | **Platform**  **(date)** | **Format** | **Strategy** | **Design (comparison)** | **Outcomes** |
| Nason et al 2015 [23]  😐 unclear risk of bias | 33 journals (including peer-reviewed articles about Urology and Nephrology) | Twitter  (6 months, November 2013 to April 2014 inclusive) | Format unspecified (except prevalence of links was measured) | Posts released by official journal Twitter accounts | Retrospective non-randomised comparison  (SM vs No SM) | Impact: Impact factor after 6 months showed a large effect in favour of journals with Twitter accounts |
| Navarro et al 2020 [24]  😐 unclear risk of bias | 236 posts (including evidence-based information and global surgery hashtags) | Twitter, Instagram  (3 months, January 2019, to March 2019) | Text posts on Twitter, media posts on Instagram and Twitter (picture/videos), and online journal clubs | Posts including # global surgery hashtags, corresponding with events (e.g., Academic Surgical Conference, Surgical Innovations and Healthcare, special edition journal release regarding global surgery by the British Journal of Surgery) and 1 journal club per month | Retrospective non-randomised comparison  (Platform vs Platform) | Reach: Views of (tagged research posts) after 3 months favoured Twitter |
| Ng et al 2020 [25]  ☺ Low risk of bias | 12 multimedia video resources (featuring guidelines based on published research about Tracheostomy safety) | Twitter, Facebook, LinkedIn, YouTube.  (12 weeks, Jan-March 2018 | Post with video (either <20 s or <2 min) with link to guidelines | Posts released daily during campaign week, 12-week paid SM campaign using Feedfirst marketing, coordinated with events and re-distributed by organisations (e.g., national tracheostomy safety week, 2018 International Tracheostomy  Symposium), involvement of key national stakeholders. | Non-randomised comparison  (SM vs no SM,  Before vs After) | Reach: Views (of YouTube video) per day showed large effects in favour of SM between events, and after,  Engagement: other Interactions (time spent watching video) after 3 months showed large effects in favour of, and after, SM  Dissemination: Guideline views and smart phone application downloads after 3 months showed a large increase after the social campaign |
| Niehaus et al 2018 [46]  😐 unclear risk of bias | 16 months of Articles (featured on SM by the Physical Medicine and Rehabilitation (PM&R) journal) | Twitter  (8 months, March 2017- October 2017) | Text Tweets with informative and additional content, links, images, increased hashtags (consistent #Physiatry plus topical, e.g., #concussion) | Posts released by journal Twitter account (@PMRJournal) twice per article, median Tweets per months rising from 14 (range, 12-16) to 36 (range, 29-57), also greater use of hashtags (consistent #Physiatry plus topical, e.g., #concussion) retweeting posts from reputable sources (e.g., institutions/government) and sharing other relevant media articles. | Observational study over 16 months  (Before vs After) | Reach: Number of followers including practitioners each month showed a large increase after the SM campaign  Engagement: Retweets (shares) after each month showed a large increase after the journal’s SM campaign  Dissemination: Article views (on journal webpage) after each month showed a large increase after the SM campaign |
| **Article /**  **Risk of Bias** | **Subjects**  **(specialism)** | **Platform**  **(date)** | **Format** | **Strategy** | **Design (comparison)** | **Outcomes** |
| O'Kelly et al, 2017 [26]  😐 unclear risk of bias | 89 Journals (that publish peer reviewed articles about paediatric urology) | Twitter, Facebook, LinkedIn, YouTube, Google+  (4 years, 2012 to 2016) | Post format not specified | Strategy not specified | Retrospective non-random comparison  (SM vs No SM,  Strategy vs Strategy) | Impact: Impact factor after 4 years showed a small effect in favour of journals with a Twitter account and in favour of journals on ≥3 SM platforms. |
| Ozkent et al, 2022 [27]  ☺ Low risk of bias | 410 Articles (published in 7 peer-reviewed journals of Sexual Medicine) | Twitter  (6 months, January 2018 to June 2018) | Post format not specified | Articles Tweeted at least once | Retrospective Non-randomised comparison  (SM vs No SM) | Impact: Citations after 2 years showed a moderate effect in favour of Tweeted articles |
| Patel et al, 2022 [28]  😐 unclear risk of bias | 31,170 Twitter users (who posted about Colorectal Cancer, posts including published research articles) | Twitter  (15 months, January 2020 and April 2021) | Text posts some with links to research articles and other relevant media | Post released by clinicians, individuals. organisations, and influencers (in this paper an influencer was defined as a user with greater than 100,000 followers) | Retrospective non-randomised comparison  (Strategy vs Strategy) | Reach: Followers including practitioners and Impressions per Tweet per user showed large and moderate effects in favour of influencers and organisations respectively |
| Reppucci et al, 2022 [29]  😐 unclear risk of bias | 247 SM accounts (with evidence-based posts about paediatric colorectal conditions) | Twitter, Facebook, Instagram  (1 month, May 2021) | Post format not specified, some including links to published research articles | Posts released in one month including topic search terms (“imperforate anus”, “anorectal malformation”, “cloaca”, “Hirschsprung disease”, “vacterl”, “cloacal exstrophy”, and “pedscolorectal”). | Retrospective non-randomised comparison  (Platform vs Platform) | Reach: number of followers after 1 month showed no significant effect between practitioner use of different platforms (Twitter, Facebook, or Instagram)  Engagement: Total interactions (number of posts, likes, tweets) after 1 month showed a large effect in favour of Twitter |
| Rohilla et al 2020 [47]  😐 unclear risk of bias | 131 Micro-blog posts (featuring clinical scenario questions with evidence-based answers about medicinal drugs) | Facebook  (6 months, August 2018, to February 2019) | Clinical questions and answers with links to research evidence | Posts released, on average, once a day. No paid SM marketing nor analytical tools. | Prospective cohort observational study  (Before vs After) | Reach: Impressions after each month showed a large increase after SM campaign  Engagement: Likes after each month showed a large increase after SM campaign |
| **Article /**  **Risk of Bias** | **Subjects**  **(specialism)** | **Platform**  **(date)** | **Format** | **Strategy** | **Design (comparison)** | **Outcomes** |
| Rotolo et al 2022 [30]  😐 unclear risk of bias | 15 infographics (featuring information about COVID-19 from trusted sources including peer-reviewed evidence) | Twitter, Instagram, Facebook, LinkedIn  (3 months March 2020, and June 2020) | Text post with 3 informal points, an infographic, hashtags #, @ mentions and emojis | Posts released, in real time as infographics were prepared, on the Illinois Medical Professionals Action Collaborative Team (IMPACT) website and associated SM accounts. | Non-randomised comparison  (Platform vs Platform) | Reach: Impressions after 7 days to 3 months (depending on when the infographic was posted) showed a large effect in favour of Twitter vs Facebook and Instagram  Engagement: Shares after 7 days to 3 months totals showed a non-significant effect in favour of Twitter vs Facebook and Instagram |
| Schwenk et al 2017 [51]  ☹ high risk of bias | 2 conferences (featuring peer-review evidence about Regional Anaesthesia and Pain Medicine) | Twitter, Facebook, Linked In  (3 days each, 2 years in May 2015 and 2016) | Text posts with short author biographies, links to research evidence, branded hashtags # and mentions @ | Post released from 6 months in advance of 2016 conference, advertised using 9 prominent American Society of Regional Anaesthesia (ASRA) members SM accounts and a branded conference hashtag #. | Case study over 2 years (Before vs After) | Reach: Number of conference participants (practitioners) and Impressions after 3 days increased with SM campaign over time |
| Segura Sampedro et al 2018 [48]  ☹ high risk of bias | 4 conferences (featuring peer-review evidence for clinicians about surgery) | Twitter  (3 days each, 4 years, from 2013 to 2016) | Posts with conference hashtags #rnc13, #cnc14, #rnc15 and #cncirugia16 respectively | Post frequency increased over time and engagement became less reliant on influencer involvement over time. | Observational over 4 years  (Before vs After) | Reach: Number of congress participants (practitioners) and Impressions after 3 days increased with SM campaign over time |
| Thoma et al 2018 [31]  😐 unclear risk of bias | 29 Articles (published in the Canadian Journal of Emergency Medicine (CJEM)) | Twitter, Facebook  (2 years, January 2015 through June 2016) | Text posts of 1-2 sentence summary, with link and either a screen capture image, infographic or podcast | Strategy not specified | Non-randomised comparison  (Format vs Format) | Dissemination: Article abstract views, Article downloads and Altmetric Attention score 1-2 years after publication showed large effects in favour of podcasts and Infographics compared to standard posts |
| Trueger et al 2018 [32]  ☺ Low risk of bias | 267 Articles (in press for original research and systematic reviews in the Annals of Emergency Medicine) | Twitter, Facebook (12-months, July 2014, to July 2015) | Text Tweets providing a brief summary or commentary, with links to article, blogs and podcasts, usually with screen capture image | Posts released, one per article, using 9 personal SM accounts belonging to the new SM team (consisting of physicians and residents with varied clinical and educational interests already active on SM) | Observational study over 18 months  (Before vs After  Platform vs Platform) | Dissemination: Article views after 6-18 months showed a moderate increase after SM exposure and a large effect in favour of Twitter compared to Facebook |
| **Article /**  **Risk of Bias** | **Subjects**  **(specialism)** | **Platform**  **(date)** | **Format** | **Strategy** | **Design (comparison)** | **Outcomes** |
| Utengen et al 2017 [33]  😐 unclear risk of bias | 1672 Conferences (registered in Symplur’s Health Care Hashtag Project) | Twitter  (2 years, 2014 to 2016) | Posts with healthcare conference hashtags # | Posts released by conferences using healthcare hashtags in at least 1000 tweets, with and without patient involvement in sharing conference evidence on SM | Retrospective comparison (Strategy vs Strategy) | Engagement: Total Tweet activity after 2 years showed a small effect in favour of patient participation with conference posts |
| Wadhwa et al 2017 [34]  ☺ Low risk of bias | 1032 Tweets (about research published in the American Journal of Neuroradiology (AJNR)) | Twitter  (12 months, August 2015 to July 2016) | Text Tweets with or without images and relevant hashtags # | Tweets released by the official AJNR Twitter account (@TheAJNR)  on weekends and weekdays, in the morning (06:00 –11:59), afternoon (12:00 –16:59), evening (17:00 –20:59), or night-time (21:00 –05:59) (Central Standard Time (CST)). | Retrospective comparison  (Format vs Format  Strategy vs Strategy) | Engagement: Total Twitter interactions per impression after 6-18 months showed large effects in favour of images, weekdays (vs weekend) and morning (vs afternoon) posts and small significant effects of relevant hashtags |
| Widmer et al 2016 [35]  😐 unclear risk of bias | 555 Tweets (about published research articles in The Mayo Clinic, about cardiology and cardiovascular medicine) | Twitter  (1 year, before 2016) | Text Tweets about publication with screenshot image with or without adjunctive synopsis or video published by a non-peer reviewed news source | Posts released, 1 to 2 times per day, using the official @MayoClinicCV Twitter account, scheduled at 8am or 4pm to 6pm Eastern Standard Time (EST). Additional ad libitum retweets, quoted tweets and modified tweets sent by account managers dependent on special circumstances such as publications, conferences and relevant events | Non-randomised comparison  (Strategy vs Strategy) | Reach: Impressions after 1 year showed a small effect in favour of adjunctive synopses or videos by non-peer reviewed sources.  Engagement: Total interactions after 1 year showed a small effect in favour of adjunctive posts by non-peer reviewed sources  Dissemination: Link clicks per tweet after 1 year showed a non-significant effect in favour of adjunctive posts by non-peer reviewed sources |
| Wray et al 2018 [36]  😐 unclear risk of bias | 15 Articles (featured in journal clubs by the Journal of Hospital Medicine (JMH)) | Twitter  (2 years October 2015 to November 2017) | Text Tweets, with visual abstracts after February 2017 | Posts released one month prior to each Tweet chat scheduled every 2-3 months (October 2015 to January 2017) and increasing in frequency to every month in 2017, at 9pm, Eastern Standard Time (EST) for 1 hour. | Non-randomised comparison (SM vs No SM Before vs After) | Dissemination: Altmetric Attention Score after 2 weeks showed a non-significant effect in favour of Tweet chat journal club featured articles and a non-significant increase after SM exposure |
| Abbreviations: SM social media | | | | | | |

Table B: Comparisons for each outcome

| **Outcome** | **Comparison** | **SMD [95% CI]** | **In favour of** | | **p value** | | **I^2^ (%)** | | **GRADE** | | **Studies and references** | |
| --- | --- | --- | --- | --- | --- | --- | --- | --- | --- | --- | --- | --- |
| Reach in number of practitioners | SM v no SM | 4.03 [3.37,4.68] | SM * | | <0.0001 | | N/A | | N/A | | 1 (1 RCT [7]) | |
|  | Before after SM | 2.03 [0.97, 3.10] | SM * | | 0.0002 | | 53 | | moderate | | 4 (4 Non RCTs [37, 40, 41, 46]) | |
|  | Platform v platform | 0.05 [-0.25, 0.35] | Twitter | | 0.74 | | N/A | | N/A | | 1 (1 non RCT [29]) | |
|  | Format v format | 0.52 [-0.23, 1.28] | Visual abstracts | | 0.17 | | N/A | | N/A | | 1 (1 RCT [3]) | |
|  | Strategy v strategy | 1.97 [1.88, 2.06] | Influencers * | | <0.0001 | | N/A | | N/A | | 1 (1 non RCT [28]) | |
| Reach in impressions or post views | SM v no SM | 2.53 [1.41,3.64] | SM * | | <0.0001 | | N/A | | N/A | | 1 (1 non RCT [25]) | |
|  | Before after SM | 1.99 [1.23, 2.75] | SM * | | <0.0001 | | 95 | | moderate | | 11 (1 RCT [7] and 10 non RCTs [15, 18, 25, 37, 38, 40, 41, 43, 45, 47]) | |
|  | Platform v platform | 1.87 [1.54, 2.21] | Twitter * | | <0.0001 | | 0 | | low | | 2 (2 non RCTs [19, 30]) | |
|  |  | 1.19 [0.64, 1.75] | Facebook * | | <0.0001 | | 46 | | low | | 2 (2 non RCTs [19, 30]) | |
|  | Format v format | 1.63 [0.04, 3.22] | Images * | | 0.04 | | 95 | | low | | 4 (2 RCTs [3, 7] and 2 non RCTs [11, 18]) | |
|  | Strategy v strategy | 1.02 [0.04, 1.99] | Influencers * | | 0.04 | | 100 | | low | | 2 (2 non RCTs [28, 35]) | |
| Engagement in positive responses | Before after SM | 3.18 [-0.25, 6.62] | SM | | 0.07 | | 98 | | low | | 3 (1 RCT [7] and 2 non RCTs [45, 47] | |
|  | Platform v platform | 1.49 [0.34, 2.64] | Twitter * | | 0.01 | | N/A | | N/A | | 1 (1 RCT [9]) | |
|  | Format v format | 0.87 [-0.40, 2.14] | Images | | 0.18 | | 88 | | low | | 2 (1 RCT [7] and 1 non RCT [11]) | |
| Engagement in interactions | SM v no SM | 2.36 [1.27, 3.44] | SM * | | <0.0001 | | N/A | | N/A | | 1 (1 non RCT [25]) | |
|  | Before after SM | 3.74 [2.02, 5.46], | SM * | | <0.0001 | | 96 | | moderate | | 7 (1 RCT [7] and 6 non-RCTs [15, 25, 37, 40, 45, 46] | |
|  | Platform v platform | 1.15 [0.21, 2.10] | Twitter * | | 0.02 | | 79 | | low | | 3 (1 RCT [9] and 2 non RCTs [29, 30]) | |
|  | Format v format | 1.24 [0.53, 1.96] | Images * | | 0.0007 | | 98 | | low | | 6 (2 RCTs [3, 7] and 4 non RCTs [11, 14, 18, 34]) | |
|  | Strategy v strategy | 5.62 [5.46, 5.79] | Influencers * | | <0.0001 | | N/A | | N/A | | 1 (1 non RCT [14]) | |
|  |  | 0.26 [0.13, 0.39] | Influential others* | | <0.0001 | | 82 | | low | | 3 (3 non RCTs [16, 33, 35]) | |
|  |  | 3.93 [3.71, 4.15] | Hashtags * | | <0.0001 | | N/A | | N/A | | 1 (1 non RCT [34]) | |
|  |  | 2.29 [2.12, 2.46] | Morning posts* | | <0.0001 | | N/A | | N/A | | 1 (1 non RCT [34]) | |
|  |  | 3.93 [3.67, 4.18] | Weekday posts* | | <0.0001 | | N/A | | N/A | | 1 (1 non RCT [34]) | |
| **Outcome** | **Comparison** | **SMD [95% CI]** | | **In favour of** | | **p value** | | **I^2^ (%)** | | **GRADE** | | **Studies and references** |
| Direct dissemination in link clicks or article views | SM v no SM | 0.88 [0.15, 1.62] | | SM * | | 0.02 | | 95 | | moderate | | 6 (6 RCTs [1, 2, 4, 5, 7, 10]) |
|  | Before after SM | 1.93 [1.23, 2.62] | | SM * | | <0.0001 | | 92 | | high | | 11 (3 RCTs [2, 6, 7] and 8 non RCTs [15, 25, 32, 41-43, 45, 46]) |
|  | Platform v platform | 0.92 [-1.21, 3.04] | | Twitter/Facebook | | 0.40 | | 99 | | low | | 2 (1 RCT [10] and 1 non RCT [32]) |
|  | Format v format | 1.18 [0.27, 2.10] | | Images * | | 0.01 | | 88 | | low | | 5 (3 RCTs [3, 6, 7] and 2 non RCTs [18, 31]) |
|  |  | 3.03 [1.55, 4.52] | | Podcast * | | <0.0001 | | N/A | | N/A | | 1 (1 non RCT [31]) |
|  | Strategy v strategy | 0.02 [-0.15, 0.20] | | News source | | 0.78 | | N/A | | N/A | | 1 (1 non RCT [35]) |
|  |  | 0.98 [0.34, 1.61] | | Tues, Wed, Sat* | | 0.002 | | N/A | | N/A | | 1 (1 non RCT [15]) |
| Direct dissemination in article downloads | SM v no SM | 1.25 [0.86, 1.65] | | SM * | | <0.0001 | | 0 | | high | | 3 (2 RCTs [2, 10] and 1 non RCT [12]) |
|  | Before after SM | 0.82 [0.26, 1.37] | | SM * | | 0.004 | | 49 | | moderate | | 5 (2 RCTs [2, 6] and 3 non RCTs [25, 41, 43]) |
|  | Format v format | 0.26 [-0.32, 0.83] | | Images | | 0.38 | | 0 | | low | | 2 (1 RCT [6] and one non RCT [31]) |
|  |  | 0.94 [-0.10, 1.98] | | Podcast | | 0.08 | | N/A | | N/A | | 1 (1 non RCT [31]) |
| Direct dissemination in altmetric score | SM v no SM | 1.48 [-1.00, 3.96] | | SM | | 0.24 | | 97 | | low | | 2 (1 RCT [7] and 1 non RCT [36]) |
|  | Before after SM | 1.92 [0.75, 3.09] | | SM | | 0.001 | | 92 | | moderate | | 7 (2 RCTs [6, 7] and 5 non RCTs [13, 36, 39, 41, 45]) |
|  | Format v format | 1.19 [0.04, 2.35] | | Images * | | 0.04 | | 83 | | low | | 3 (2 RCTs [6, 7] and 1 non RCT [31]) |
|  |  | 3.28 [1.72, 4.84] | | Podcast * | | <0.0001 | | N/A | | N/A | | 1 (1 non RCT [31]) |
| Impact in citations or impact factor | SM v no SM | 0.76 [0.49, 1.03] | | SM * | | <0.0001 | | 79 | | moderate | | 8 (2 RCTs [8, 10] and 6 non RCTs [17, 20, 21, 23, 26, 27]) |
|  | Platform v platform | 0.77 [0.34, 1.21] | | ≥ 3 platforms * | | 0.0005 | | N/A | | N/A | | 1 (1 non RCT [26]) |
|  | Strategy v strategy | 1.00 [-0.84, 2.84] | | Author tweets | | 0.29 | | 98 | | very low | | 2 (2 non RCTs [16, 17]) |
| Impact on thinking or practice | SM v no SM | 0.65 [0.37, 0.93] | | SM * | | <0.0001 | | 55 | | low | | 2 (2 non RCTs [19, 22]) |
|  | Before after SM | 0.45 [0.07, 0.83] | | SM * | | 0.02 | | 91 | | low | | 3 (1 RCT [9] and 2 non RCTs [22, 44]) |
|  | Platform v platform | 0.24 [-0.05, 0.53] | | Twitter | | 0.10 | | N/A | | N/A | | 1 (1 RCT [9]) |
| Abbreviations: SMD standardised mean difference effect size (Hedges g), CI confidence intervals, I^2^ dispersion measure of heterogeneity, GRADE Grading of Recommendations Assessment, Development and Evaluation, SM social media, v versus, N/A not applicable, RCT randomised controlled trial, * indicates significant effect | | | | | | | | | | | | |

## Reach

Table B summarises reach between and within group effectiveness of social media. In addition, two small studies, both with high risk of bias, whose data were not suitable for meta-analysis, showed increased reach in conference research evidence sharing after the introduction of social media strategies compared to before [48, 51].

Platform

One observational study [29], with moderate risk of bias, indicated no significant difference in the number of practitioners who followed healthcare information on different social media platforms (Twitter, Facebook and Instagram).

Pooled data for impressions from two studies [19, 30] indicated a large, significant effect size in favour of Twitter (compared to Instagram [19, 30] and Facebook [19]) (1.87 [1.54, 2.21], p<0.0001, I^2^=0%, Figure A).

Figure A: Meta-analysis of the effect of Twitter vs Instagram or Facebook on reach in impressions or post views


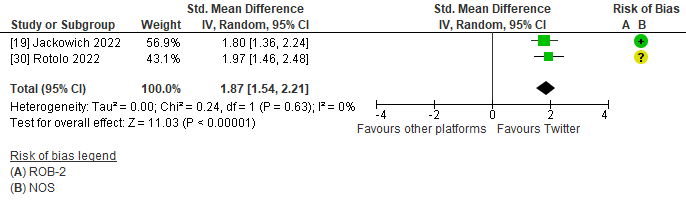


Pooled effects from two studies [19, 30] also showed a large significant effect in favour of Facebook compared to Instagram (1.19 [0.64, 1.75], <0.0001, I^2^=46%) (Figure B).

Figure B: Meta-analysis of the effect of Facebook vs Instagram on reach in impressions or post views


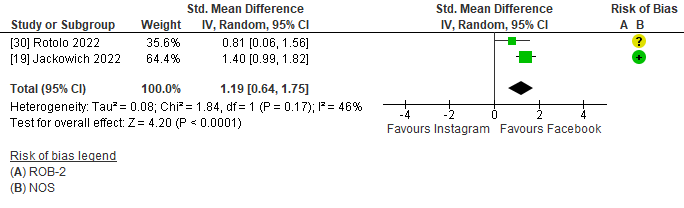


Another study [24], with moderate risk of bias, whose data were not suitable for meta-analysis, compared the number of tagged research posts between platforms and indicated a large effect in favour of Twitter vs Instagram.

Format

One RCT of 41 surgical articles [3], with low risk of bias, indicated a moderate, non-significant, effect of format on the number of practitioners reached in favour of visual abstracts compared to standard posts or plain language summaries (0.52 [-0.23, 1.28], p=0.17).

Pooled data from four studies (two RCTs of 112 [7] and 41 [3] articles, one crossover [18] and one observational study [11]) indicated a large significant effect of format on impressions in favour of images compared to standard posts (1.63 [0.04, 3.22], p=0.04, I^2^=95%). The two RCTs indicated a moderate, but non-significant, effect of format on impressions in favour of posts with images (photos [7] or visual abstracts [3]) (0.42 [-0.18, 1.03], p=0.17, I^2^=43%). Whereas there were significant large [11] and very large [18] effects of format on impressions in favour of posts with infographics (1.92 [1.16, 2.69] and 3.65 [2.96, 4.35], p<0.00001) (Figure C).

Figure C: Meta-analysis of the effect of image vs no image on reach in impressions


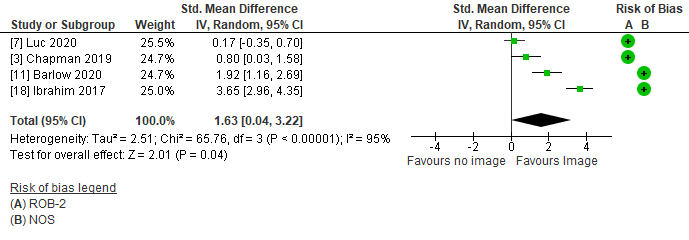


A case study [50], with high risk of bias, whose data were not suitable for meta-analysis, of a single geriatrics research article also showed increased reach in impressions for an infographic compared to a standard Tweet.

Strategy

Pooled data from two studies [28, 35] indicated a large significant effect of influencers (including organisations and non-peer-reviewed news sources) on impressions (1.02 [0.04, 1.99], p=0.04, I^2^=100%). One non-randomised study [28] indicated a large effect on reach of influencers in (followers 1.97 [1.88, 2.06] and impressions 1.99 [1.90, 2.08], both p<0.00001) and a moderate effect of organisations (followers 0.58 [0.55, 0.60] and impressions 0.65 [0.62, 0.67], both p<0.0001). The other non-randomised study [35] indicated a small effect on reach in impressions of non-peer reviewed news sources sharing research evidence (0.41 [0.23, 0.58], p<0.0001) (Figure D).

Figure D: Meta-analysis of the effect of influencer or organisation on reach in impressions or views


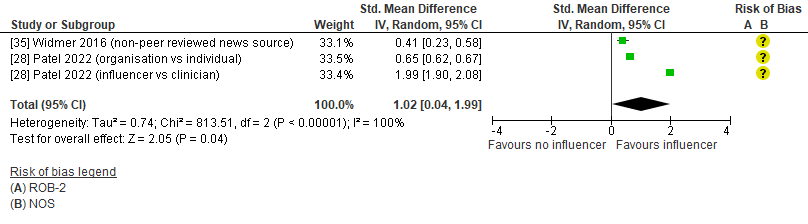


Another observational study [13], with high risk of bias, whose data were unsuitable for meta-analyses, also indicated a large effect on impressions of a single, extremely popular, influencer account compared to standard Twitter accounts.

Finally, one RCT follow-up study [8], with low risk of bias, indicated that Tweeting at 1 pm (EST, USA) generated the highest and 9 pm generated the lowest, reach to physicians (p=0.004) and to the public (p=0.022).

## Engagement

Table B summarises engagement between and within group effectiveness of social media. In addition, one case study, with high risk of bias, whose findings that were not suitable for meta-analysis, suggested significantly improved engagement after a social media strategy to share one article [49].

Platform

One RCT [9], with high risk of bias, indicated a large significant effect on likes between platforms (in favour of Twitter vs Facebook) (1.49 [0.34, 2.64], p=0.01).

Pooled data from the RCT [9] and two observational studies [29, 30] indicated a large significant effect on interactions (replies, shares, retweets or total interactions) in favour of Twitter (vs Facebook, Instagram and LinkedIn) (1.15 [0.21, 2.10], p=0.02, I^2^=79%). The largest effect in favour of Twitter (vs Facebook) was in the RCT [9]. Whereas the two non-randomised studies [29, 30] indicated a more moderate significant effect in favour of Twitter (0.73 [0.23, 1.24], p=0.004, I^2^=48%) (Figure E).

Figure E: Meta-analysis of the effect of Twitter vs Facebook, Instagram or LinkedIn on engagement in interactions


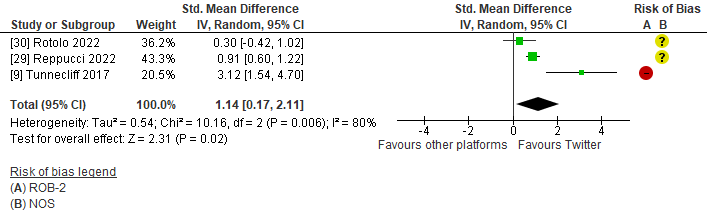


Format

Pooled data from one RCT [7] and one observational study [11] indicated a large, non-significant, effect of images on likes (0.87 [-0.40, 2.14], p=0.18, I^2^=88%). The RCT of 112 articles [7] indicated a small, non-significant, effect of format on likes in favour of posts with photos (including screen shots of journal articles) (vs no photos). The observational study of 40 evidence-based tweets [11] indicated a large significant effect of format on likes in favour of infographics (vs Tweets with links) (Figure F).

Figure F: Meta-analysis of the effect of image vs no image on engagement in likes


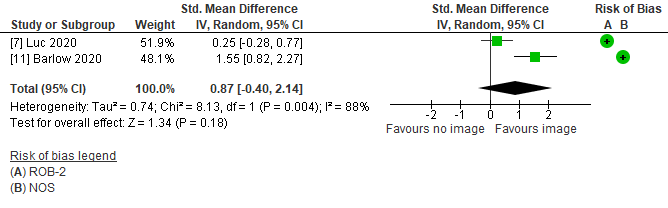


Pooled data from two RCTs [3, 7], one crossover study [18] and three observational studies [11, 14, 34] indicated a large significant effect of format in favour of images (vs no images) on interactions (1.24 [0.53, 1.96], p=0.0007, I^2^=98%). The largest effect sizes in favour of images were for studies featuring infographics [11, 18], then unspecified embedded images [34]. Small, significant effects in favour of images vs no image were indicated by findings from two RCTs (one that compared infographics and article screenshot images vs plain language summaries [3] and the other that compared posts with photos vs no photos [7]) and one observational study (that compared standard tweets with those including images or video media [14]) (Figure G).

Figure G: Meta-analysis of the effect of image vs no image on engagement interactions


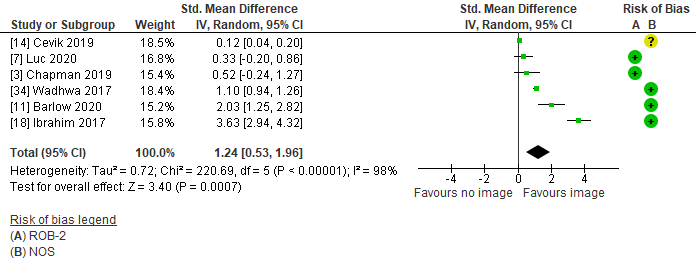


A case study [50], with high risk of bias, whose data were not suitable for meta-analysis, showed increased engagement with an infographic compared to a standard Tweet for one geriatrics research article.

Strategy

One observational study [14], with low risk of bias, comparing Twitter accounts with >1000 vs <1000 followers indicated a large significant effect size of social media influencers on post interactions (5.62 [5.46, 5.79], p<0.00001).

Pooled findings from three observational studies [16, 33, 35] indicated a small significant effect on post interactions of other influences (patient participation in conference social media [33], adjunctive non-peer reviewed news source involvement [35] and authors who tweeted about their articles [16]) (0.26 [0.13, 0.39], p<0.0001, I^2^=82%) (figure H).

Figure H: Meta-analysis of the effect of other influences on engagement in interactions


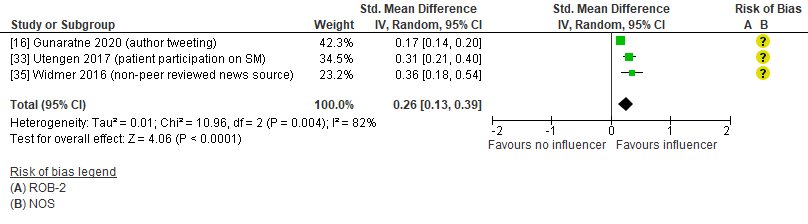


One observational study [34], with low risk of bias, indicated large effects on post interactions of hashtags vs no hashtags (3.93 [3.71, 4.15], p<0.00001), morning vs afternoon posts (central daylight time, USA) (2.29 [2.12, 2.46], p<0.00001) and weekday vs weekend posts (3.93 [3.67, 4.18], p<0.00001).

One observational study [14], with moderate risk of bias, indicated a small, significant effect of @ mentions or tags compared to standard Tweets on the number of retweets (0.23 [0.16, 0.30], p<0.00001).

## Direct Dissemination

Table B summarises direct dissemination between and within group effectiveness of social media. In addition, one case study [49], with high risk of bias, whose findings were not suitable for meta-analysis, showed between and within group effects on article views and altmetric attention score of social media used to promote an article compared to predicted dissemination without social media.

Platform

Pooled data showed no significant between group effect of platform on direct dissemination (0.92 [-1.21, 3.04], p=0.40, I^2^=99%). The RCT [10] indicated a large, significant effect on article views in favour of Facebook (vs Twitter) (1.00 [1.48, 0.53], p<0.0001) and Twitter (vs LinkedIn) (1.27 [0.74, 1.79], p<0.00001). Conversely, the observational study [32] indicated a large significant effect on article views in favour of Twitter (vs Facebook) (2.46 [2.24, 2.69], p<0.00001)) (Figure J).

Figure J: Meta-analysis of the effect of Twitter vs Facebook and LinkedIn on article views


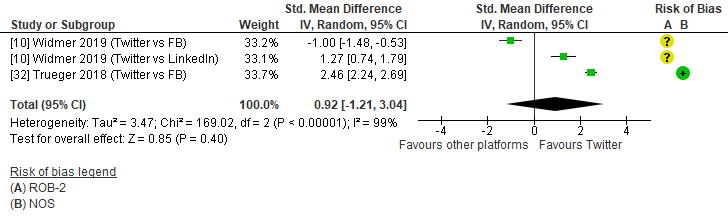


Format

Pooled data from three RCTs [3, 6, 7], one crossover study [18] and one non-randomised intervention study [31] indicated a large, significant effect of format on link clicks or article views in favour of images vs no images (1.18 [0.27, 2.10], p=0.01, I^2^=88%). The crossover study [18] reported the largest effect size in favour of infographics (vs article title posts). Similarly, the non-randomised study reported a large significant effect size in favour of infographics [31]. Small [3], moderate [7] and large [6] effect sizes of visual abstracts, photos and infographics respectively were indicated by findings in the RCTs (Figure K).

Figure K: Meta-analysis of the effect of image vs no image on article link clicks or views


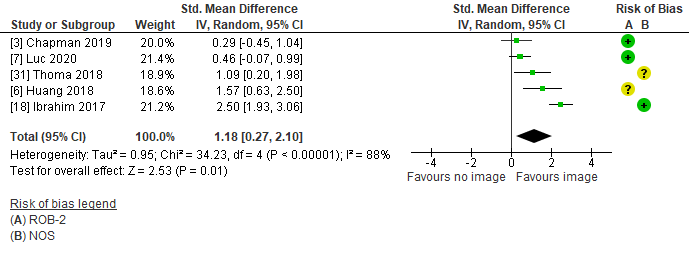


Pooled data from one RCT [6] and one non-randomised study [31] indicated a small, non-significant, effect on PDF article downloads of format in favour of image compared to no image (0.26 [-0.32, 0.83], p=0.38, I^2^=0%) (Figure L).

Figure L: Meta-analysis of the effect of image vs no image on PDF article downloads


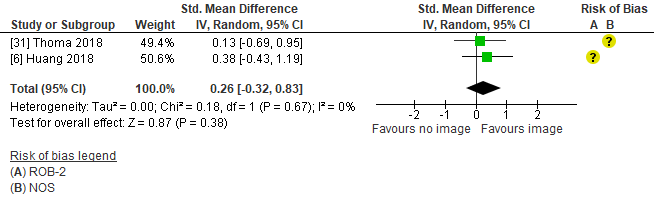


Pooled data from two RCTs [6, 7] and one non-randomised comparison study [31] indicated a large significant effect of format on altmetric attention score in favour of images compared to no image (1.19 [0.04, 2.35], p=0.04, I^2^=83%). Large significant effects of infographics were reported by the non-randomised study [31] and the RCT [6] with unclear risk of bias. Whereas the RCT [7] with low risk of bias indicated a small, non-significant, effect of posts with photos compared to posts without photos (Figure M).

Figure M: Meta-analysis of the effect of image vs no image on altmetric attention score


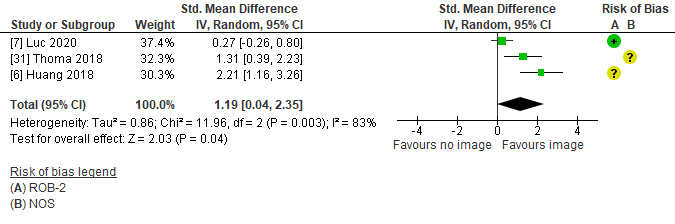


One observational study [31], with moderate risk of bias, demonstrated large effects of format in favour of podcasts compared to standard posts for dissemination. Effects were significant for article views (3.03 [1.55, 4.52], p<0.00001) and altmetric attention score (3.28 [1.72, 4.84], p<0.00001) but not for PDF downloads (0.94 [-0.10, 1.98], p=0.08). Effects were larger than the effect sizes for infographics compared to standard posts in the same study.

One non-randomised comparison [11], with low risk of bias, indicated a moderate, non-significant effect of format on link clicks in favour of posts with links compared to posts with infographics (-0.56 [-1.20, 0.08], p=0.09).

Strategy

One observational study [15], with moderate risk of bias, indicated a large significant effect of the day of the week on link clicks in favour of Tuesdays, Wednesdays and Saturdays compared to the other days of the week (0.98 [0.34, 1.61], p=0.002).

Two RCTs [4, 5], with low risk of bias, indicated that time of year did not appear to have an effect on article views.

One observational study [35], with moderate risk of bias, indicated that posts with adjunctive non-peer reviewed news source influencer involvement did not have an effect on link clicks (0.02 [-0.15, 0.20], p=0.78).

## Impact

Table B summarises impact between and within group effectiveness of social media. In addition, one non-randomised study [36], with moderate risk of bias, whose impact data were not suitable for meta-analysis, reported within group impact on practice after participation in a Tweet chat; changes included changing personal practice, teaching others about the new practice, revising a protocol or institutional policy or procedure, or educating patients about the new practice.

Platform

No included studies compared platforms in terms of citations. However, two observational studies [21, 26], both with moderate risk of bias, indicated that journals sharing research on a greater number of social media platforms had higher impact factors (one by comparison of ≥ 3 platforms with < 3 platforms (0.77 [0.34, 1.21], p=0.0005) [26] the other by association [21]).

One RCT [9] with high risk of bias indicated a small, non-significant effect on knowledge and practice in favour of Twitter (vs Facebook) for delivering evidence-based tendinopathy practice points via social media (0.24 [-0.05, 0.53], p=0.10).

Format

No included studies compared post format effects on impact.

Strategy

Pooled data from two studies [16, 17] indicated a large, but not significant, effect on citations of author Tweeting (1.00 [-0.84, 2.84], p=0.29, I^2^=98%). One observational study [17] indicated a large, significant effect on citations of authors Tweeting in addition to the journal social media strategy (1.96 [1.45, 2.46], p<0.00001). The other observational study [16] indicated a negligible, but significant effect on citations of author Tweeting (0.08 [0.05, 0.11], p<0.00001) (Figure N).

Figure N: Meta-analysis of the effect of author Tweets vs no author Tweets on citations


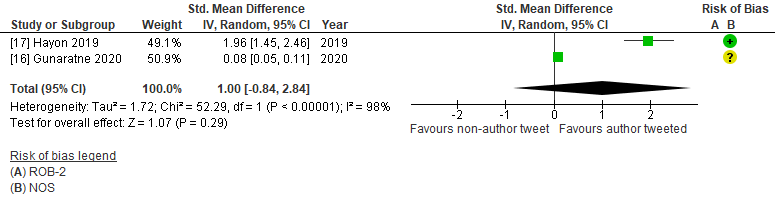


## References

[1] C. E. Adams *et al.*, “Tweeting links to Cochrane Schizophrenia Group reviews: a randomised controlled trial,” *BMJ Open,* vol. 6, no. 3, pp. e010509, Mar 08, 2016.

[2] H. G. Allen *et al.*, “Social media release increases dissemination of original articles in the clinical pain sciences,” *PLoS One,* vol. 8, no. 7, pp. e68914, 2013.

[3] S. J. Chapman *et al.*, “Randomized controlled trial of plain English and visual abstracts for disseminating surgical research via social media,” *Br J Surg*, pp. 1611-1616, Oct 02, 2019.

[4] C. S. Fox *et al.*, “A randomized trial of social media from Circulation,” *Circulation,* vol. 131, no. 1, pp. 28-33, Jan 06, 2015.

[5] C. S. Fox *et al.*, “Randomized Controlled Trial of Social Media: Effect of Increased Intensity of the Intervention,” *J Am Heart Assoc,* vol. 5, no. 5, Apr 27, 2016.

[6] S. Huang *et al.*, “The effect of an infographic promotion on research dissemination and readership: A randomized controlled trial,” *CJEM,* vol. 20, no. 6, pp. 826-833, Nov, 2018.

[7] J. G. Y. Luc *et al.*, “Social Media Improves Cardiothoracic Surgery Literature Dissemination: Results of a Randomized Trial,” *Ann Thorac Surg,* vol. 109, no. 2, pp. 589-595, Feb, 2020.

[8] J. G. Y. Luc *et al.*, “Does Tweeting Improve Citations? One-Year Results From the TSSMN Prospective Randomized Trial,” *Ann Thorac Surg,* vol. 111, no. 1, pp. 296-300, Jan, 2021.

[9] J. Tunnecliff *et al.*, “Translating evidence to practice in the health professions: a randomized trial of Twitter vs Facebook,” *Journal of the American Medical Informatics Association,* vol. 24, no. 2, pp. 403-408, 2017.

[10] R. J. Widmer *et al.*, “Effect of Promotion via Social Media on Access of Articles in an Academic Medical Journal: A Randomized Controlled Trial,” *Acad Med,* vol. 94, no. 10, pp. 1546-1553, Oct, 2019.

[11] B. Barlow *et al.*, “"Capturing your audience": analysis of Twitter engagements between tweets linked with an educational infographic or a peer-reviewed journal article,” *Journal of Visual Communication in Medicine,* vol. 43, no. 4, pp. 177-183, 2020.

[12] N. Botting, L. Dipper, and K. Hilari, “The effect of social media promotion on academic article uptake,” *Journal of the Association for Information Science & Technology,* vol. 68, no. 3, pp. 795-800, 2017.

[13] K. A. Cawcutt *et al.*, “Use of a Coordinated Social Media Strategy to Improve Dissemination of Research and Collect Solutions Related to Workforce Gender Equity,” *Journal of Women's Health,* vol. 28, no. 6, pp. 849-862, 2019.

[14] M. Cevik, D. S. Y. Ong, and G. Mackenzie, “How scientists and physicians use Twitter during a medical congress,” *Clinical microbiology and infection : the official publication of the European Society of Clinical Microbiology and Infectious Diseases,* vol. 25, no. 12, pp. 1561.e7-1561.e12, 2019.

[15] K. M. Fargen *et al.*, “Expanding the social media presence of the Journal of Neurointerventional Surgery: editor's report,” *Journal of neurointerventional surgery,* vol. 9, no. 2, pp. 215-218, 2017.

[16] K. Gunaratne, H. Haghbayan, and E. A. Coomes, “Tweeting Authors: Impact on Research Publicity and Downstream Citations,” *J Gen Intern Med,* vol. 35, no. 6, pp. 1926-1927, Jun, 2020.

[17] S. Hayon *et al.*, “Twitter Mentions and Academic Citations in the Urologic Literature,” *Urology,* vol. 123, pp. 28-33, Jan, 2019.

[18] A. M. Ibrahim *et al.*, “Visual Abstracts to Disseminate Research on Social Media: A Prospective, Case-control Crossover Study,” *Ann Surg,* vol. 266, no. 6, pp. e46-e48, Dec, 2017.

[19] R. A. Jackowich *et al.*, “#PGADFacts: Results from a 12-month knowledge translation campaign on persistent genital arousal disorder/genito-pelvic dysesthesia (PGAD/GPD),” *Canadian Journal of Human Sexuality,* vol. 31, no. 2, pp. 242-252, 2022.

[20] J. W. Jeong *et al.*, “The impact of social media on citation rates in coloproctology,” *Colorectal Disease,* vol. 21, no. 10, pp. 1175-1182, 2019.

[21] S. Mobarak *et al.*, “The importance of social media to the academic surgical literature: Relationship between Twitter activity and readership metrics,” *Surgery,* vol. 170, no. 3, pp. 650-656, 2021.

[22] P. Narayanaswami *et al.*, “The Impact of Social Media on Dissemination and Implementation of Clinical Practice Guidelines: A Longitudinal Observational Study,” *Journal of Medical Internet Research,* vol. 17, no. 8, pp. e193, 2015.

[23] G. J. Nason *et al.*, “The emerging use of Twitter by urological journals,” *BJU Int,* vol. 115, no. 3, pp. 486-90, Mar, 2015.

[24] S. M. Navarro *et al.*, “Identifying New Frontiers for Social Media Engagement in Global Surgery: An Observational Study,” *World journal of surgery,* vol. 44, no. 9, pp. 2881-2891, 2020.

[25] F. K. Ng *et al.*, “From smartphone to bed-side: exploring the use of social media to disseminate recommendations from the National Tracheostomy Safety Project to front-line clinical staff,” *Anaesthesia,* vol. 75, no. 2, pp. 227-233, 2020.

[26] F. O'Kelly *et al.*, “The effect of social media (#SoMe) on journal impact factor and parental awareness in paediatric urology,” *Journal of pediatric urology,* vol. 13, no. 5, pp. 513.e1-513.e7, 2017.

[27] M. S. Ozkent *et al.*, “Correlation between Twitter mentions and academic citations in sexual medicine journals,” *International journal of impotence research,* vol. 34, no. 6, pp. 593-598, 2022.

[28] V. R. Patel *et al.*, “#ColonCancer: Social Media Discussions About Colorectal Cancer During the COVID-19 Pandemic,” *JCO Clinical Cancer Informatics,* vol. 6, pp. e2100180, 2022.

[29] M. L. Reppucci *et al.*, “The use of social media among the pediatric colorectal community,” *Pediatric Surgery International,* vol. 38, no. 1, pp. 43-50, 2022.

[30] S. M. Rotolo *et al.*, “A coordinated strategy to develop and distribute infographics addressing COVID-19 vaccine hesitancy and misinformation,” *Journal of the American Pharmacists Association : JAPhA,* vol. 62, no. 1, pp. 224-231, 2022.

[31] B. Thoma *et al.*, “The impact of social media promotion with infographics and podcasts on research dissemination and readership,” *CJEM Canadian Journal of Emergency Medical Care,* vol. 20, no. 2, pp. 300-306, 2018.

[32] N. S. Trueger *et al.*, “Impact of a Physician-Led Social Media Sharing Program on a Medical Journal's Web Traffic,” *Journal of the American College of Radiology,* vol. 15, no. 1 Pt B, pp. 184-189, 2018.

[33] A. Utengen *et al.*, “Patient Participation at Health Care Conferences: Engaged Patients Increase Information Flow, Expand Propagation, and Deepen Engagement in the Conversation of Tweets Compared to Physicians or Researchers,” *Journal of Medical Internet Research,* vol. 19, no. 8, pp. e280, 2017.

[34] V. Wadhwa *et al.*, “Maximizing the Tweet Engagement Rate in Academia: Analysis of the AJNR Twitter Feed,” *Ajnr: American Journal of Neuroradiology,* vol. 38, no. 10, pp. 1866-1868, 2017.

[35] R. Widmer *et al.*, “An academic healthcare Twitter account: The Mayo Clinic experience,” *Cyberpsychology, Behavior, and Social Networking,* vol. 19, no. 6, pp. 360-366, Jun, 2016.

[36] C. M. Wray, A. D. Auerbach, and V. M. Arora, “The Adoption of an Online Journal Club to Improve Research Dissemination and Social Media Engagement Among Hospitalists,” *Journal of Hospital Medicine (Online),* vol. 13, no. 11, pp. 764-769, 2018.

[37] A. Bolderston *et al.*, “Five years of #MedRadJClub: An impact evaluation of an established twitter journal club,” *Journal of Medical Radiation Sciences,* vol. 69, no. 2, pp. 165-173, 2022.

[38] S. Carley *et al.*, “Social-media-enabled learning in emergency medicine: a case study of the growth, engagement and impact of a free open access medical education blog,” *Postgraduate Medical Journal,* vol. 94, no. 1108, pp. 92-96, 2018.

[39] M. P. Dyson *et al.*, “Social Media for the Dissemination of Cochrane Child Health Evidence: Evaluation Study,” *Journal of Medical Internet Research,* vol. 19, no. 9, pp. e308, 2017.

[40] A. I. Gardhouse *et al.*, “#GeriMedJC: The Twitter Complement to the Traditional-Format Geriatric Medicine Journal Club,” *Journal of the American Geriatrics Society,* vol. 65, no. 6, pp. 1347-1351, 2017.

[41] A. Gates *et al.*, “Dissemination of evidence in paediatric emergency medicine: a quantitative descriptive evaluation of a 16-week social media promotion,” *BMJ open,* vol. 8, no. 6, pp. e022298, 2018.

[42] C. M. Hawkins *et al.*, “The impact of social media on readership of a peer-reviewed medical journal,” *Journal of the American College of Radiology,* vol. 11, no. 11, pp. 1038-43, 2014.

[43] J. K. Hoang *et al.*, “Using Social Media to Share Your Radiology Research: How Effective Is a Blog Post?,” *J Am Coll Radiol,* vol. 12, no. 7, pp. 760-5, Jul, 2015.

[44] S. Maloney *et al.*, “Translating Evidence Into Practice via Social Media: A Mixed-Methods Study,” *Journal of Medical Internet Research,* vol. 17, no. 10, pp. e242, 2015.

[45] P. McNamara, and K. Usher, “Share or perish: Social media and the International Journal of Mental Health Nursing,” *International Journal of Mental Health Nursing,* vol. 28, no. 4, pp. 960-970, 2019.

[46] W. N. Niehaus, J. K. Silver, and M. S. Katz, “The PM&R Journal Implements a Social Media Strategy to Disseminate Research and Track Alternative Metrics in Physical Medicine and Rehabilitation,” *Pm & R,* vol. 10, no. 5, pp. 538-543, 2018.

[47] R. Rohilla *et al.*, “Appropriateness evaluation of Drug Information Center's Facebook page for evidence-based drug information dissemination,” *Journal of the American Pharmacists Association: JAPhA,* vol. 60, no. 6, pp. e129-e132, 2020.

[48] J. J. Segura Sampedro *et al.*, “Twitter ® use and its implications in Spanish Association of Surgeons meetings and congresses,” *Cirugia espanola,* vol. 96, no. 6, pp. 352-356, 2018.

[49] E. H. Buckarma *et al.*, “Influence of Social Media on the Dissemination of a Traditional Surgical Research Article,” *J Surg Educ,* vol. 74, no. 1, pp. 79-83, 2017.

[50] L. A. Lindquist, and V. Ramirez-Zohfeld, “Visual Abstracts to Disseminate Geriatrics Research Through Social Media,” *Journal of the American Geriatrics Society,* vol. 67, no. 6, pp. 1128-1131, 2019.

[51] E. S. Schwenk *et al.*, “Upgrading a Social Media Strategy to Increase Twitter Engagement During the Spring Annual Meeting of the American Society of Regional Anesthesia and Pain Medicine,” *Regional Anesthesia & Pain Medicine,* vol. 42, no. 3, pp. 283-288, 2017.
